# Applying machine learning techniques to predict the risk of distant metastasis from gastric cancer: a real world retrospective study

**DOI:** 10.3389/fonc.2024.1455914

**Published:** 2024-12-05

**Authors:** Xinxin Qin, Binxu Qiu, Litao Ge, Song Wu, Yuye Ma, Wei Li

**Affiliations:** ^1^ Department of Gastric and Colorectal Surgery, General Surgery Center, The First Hospital of Jilin University, Changchun, China; ^2^ Nanjing Luhe People’s Hostipal, General Surgery, Nanjing, China

**Keywords:** gastric cancer, distant metastasis, machine learning, web calculator, external validation

## Abstract

**Background:**

Distant metastasis of gastric cancer can seriously affect the treatment strategy of gastric cancer patients, so it is essential to identify patients at high risk of distant metastasis of gastric cancer earlier.

**Method:**

In this study, we retrospectively collected research data from 18,472 gastric cancer patients from the SEER database. We applied six machine learning algorithms to construct a model that can predict distant metastasis of gastric cancer. We constructed the machine learning model using 10-fold cross-validation. We evaluated the model using the area under the receiver operating characteristic curves (AUC), the area under the precision-recall curve (AUPRC), decision curve analysis, and calibration curves. In addition, we used Shapley’s addition interpretation (SHAP) to interpret the machine learning model. We used data from 1595 gastric cancer patients in the First Hospital of Jilin University for external validation. We plotted the correlation heat maps of the predictor variables. We selected an optimal model and constructed a web-based online calculator for predicting the risk of distant metastasis of gastric cancer.

**Result:**

The study included 18,472 patients with gastric cancer from the SEER database, including 4,202 (22.75%) patients with distant metastases. The results of multivariate logistic regression analysis showed that age, race, grade of differentiation, tumor size, T stage, radiotherapy, and chemotherapy were independent risk factors for distant metastasis of gastric cancer. In the ten-fold cross-validation of the training set, the average AUC value of the random forest (RF) model was 0.80. The RF model performed best in the internal test set and external validation set. The RF model had an AUC of 0.80, an AUPRC of 0.555, an accuracy of 0.81, and a precision of 0.78 in the internal test set. The RF model had a metric AUC of 0.76 in the external validation set, an AUPRC of 0.496, an accuracy of 0.82, and a precision of 0.81. Finally, we constructed a network calculator for distant metastasis of gastric cancer using the RF model.

**Conclusion:**

With the help of pathological and clinical indicators, we constructed a well-performing RF model for predicting the risk of distant metastasis in gastric cancer patients to help clinicians make clinical decisions.

## Introduction

Gastric cancer is one of the common malignant tumors in the digestive tract. According to the global cancer incidence and mortality statistics in 2020, there are more than 1 million new cases of gastric cancer and about 768,000 deaths (1). In recent years, although the incidence and mortality rates of gastric cancer have decreased, the detection rate and prognosis are not satisfactory (2). It has increasingly become one of the significant public health problems threatening human health. The 5-year overall survival rate of patients with limited early gastric cancer is usually high (>60%). In contrast, the 5-year overall survival rates of patients with locally and distantly metastasized gastric cancer have dropped dramatically to 30% and 5%, respectively (3). Several related studies have shown that early diagnosis and early treatment for people at high risk of developing distant metastases will improve the outcome and prognosis of gastric cancer patients. For early gastric cancer patients, surgical resection is the best treatment choice; for patients who cannot undergo surgical resection or advanced metastasis, chemotherapy is the most essential treatment method (4, 5). Traditionally, the prediction of tumor outcomes and treatment recommendations for gastric cancer has been mainly determined by the tumor-lymph node metastasis (TNM) staging system (6). However, given the wide variation in clinical outcomes even within the same stage group, there is a need to construct a more detailed and personalized prediction model. By combining relevant clinicopathological indicators, such as age, gender, tumor size, presence of infiltration, and adjuvant radiotherapy, these correlates have been shown to provide more individualized information about predicting distant metastasis in gastric cancer (7–11).

Artificial Intelligence (AI) is a field of computer science dedicated to creating intelligent machines capable of performing tasks requiring human intelligence (12). The core of AI includes machine learning, deep learning, natural language processing, and perceptual technologies, which give AI the ability to make autonomous decisions and understand language and visual perception. A key advantage of AI in medical forecasting is its ability to process and analyze large amounts of complex data quickly and accurately (13). Machine learning is a systematic process of learning and training from data and accurately predicting the occurrence of future events, and it is a subfield of artificial intelligence (14). Compared to traditional prediction models, machine learning-based models have several advantages, including the ability to handle large, complex data sets, identify non-linear relationships, and improve prediction accuracy. Machine learning algorithms can combine multiple variables to develop accurate and personalized predictions (15, 16). The basic idea of machine learning is to use data and statistical analysis to build models that make predictions or automated decisions by extracting patterns, relationships, and trends from data. For example, in the medical field, machine learning can diagnose diseases from medical images and provide decision support to doctors.

In this study, we investigated the factors affecting distant metastasis of gastric cancer by utilizing some common clinical indicators and related pathological factors combined with corresponding machine learning methods and theories. We constructed a prediction model for distant metastasis of gastric cancer and utilized the best-performing prediction model to predict the risk of distant metastasis of gastric cancer, which provides a sufficient basis for clinicians’ clinical decisions.

## Materials and methods

### Patient cohort

**Figure 1:**
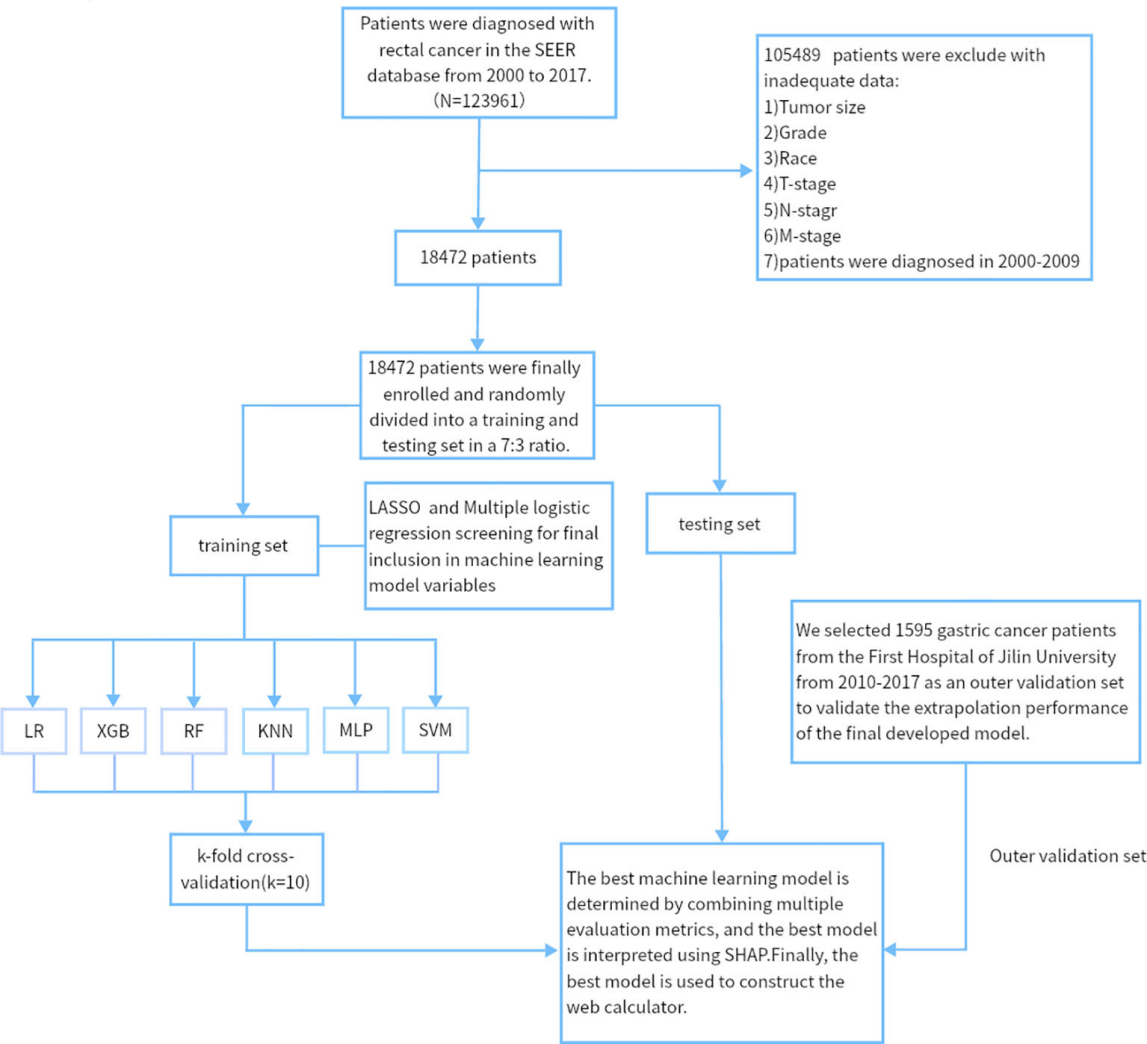
The workflow diagram for study design and patient screening. SEER, The Surveillance, Epidemiology, and End Results; LR, Logistic regression; MLP, Multilayer perceptron; XGB, The extreme gradient boosting machine; RF, Random Forest; SVM, Support vector machine; KNN, K-nearest neighbor; SHAP, Shapley’s Additive explanations.

We retrospectively analyzed data from nearly 1595 patients admitted to the First Hospital of Jilin University with the SEER database. The Surveillance, Epidemiology, and End Results (SEER) database is a publicly available, federally funded cancer reporting system that contains information from 18 states representing all regions of the country (17). By accessing the SEER database, we created a gastric cancer cohort using information about gastric cancer patients in the database. For more information about the SEER database, which does not contain sensitive content or patient-identifying information, you can visit its official website (http://seer.cancer.gov/about/), so these data can be used without approval from the ethics committee. The inclusion criteria for the gastric cancer cohort in this study were (1) a primary pathological diagnosis of gastric cancer, (2) gastric cancer as their primary tumor, (3) diagnosis between 2010 and 2017, and (4) patients with complete clinical information, including age, gender, race, marriage, pathological grading, tumor size, T stage, N stage, radiotherapy, chemotherapy, and primary site. We used data from 1,801 gastric cancer patients diagnosed at the First Hospital of Jilin University from 2010 to 2017 as an external validation cohort. Inclusion criteria for the external validation set were (1) heterochronic liver metastases (post-diagnosis) and (2) patients who did not receive preoperative neoadjuvant therapy. The study was retrospective, had no patient safety or privacy implications, and was granted an ethical waiver. Specific information regarding the SEER and external validation gastric cancer cohorts is displayed in **Tables 1**–**3**. The study flow of this paper is shown in **Figure 1**.

**Table 1:** Clinical and pathological characteristics of the study population for the training set.

Variables	DM (-)	DM (+)	P value
	9960	2970	
Age, n (%)			
≤50	935 (9.4)	428 (14.4)	P<0.001
>50	9025 (90.6)	2542 (85.6)	
Sex, n (%)			
Male	3853 (38.7)	1048 (35.3)	P=0.001
Female	6107 (61.3)	1922 (64.7)	
Race, n (%)			
White	6762 (67.9)	2136 (71.9)	P<0.001
Black	1268 (12.7)	374 (12.6)	
Other	1930 (19.4)	460 (15.5)	
Marital, n (%)			
Married	5803 (58.3)	1715 (57.7)	P=0.001
Single	1380 (13.9)	488 (16.4)	
Other	2777 (27.9)	767 (25.8)	
Grade, n (%)			
Grade I	1402 (14.1)	92 (3.1)	P<0.001
Grade II	2912 (29.2)	696 (23.4)	
Grade III	5376 (54.0)	2068 (69.6)	
Grade IV	270 (2.7)	114 (3.8)	
T stage, n (%)			
T1	2780 (27.9)	539 (18.1)	P<0.001
T2	1609 (16.2)	191 (6.4)	
T3	3335 (33.5)	623 (21.0)	
T4	1780 (17.9)	872 (29.4)	
Tx	456 (4.6)	745 (25.1)	
N stage, n (%)			
N0	5214 (52.3)	984 (33.1)	P<0.001
N1	2042 (20.5)	1071 (36.1)	
N2	1205 (12.1)	269 (9.1)	
N3	1238 (12.4)	356 (12.0)	
Nx	261 (2.6)	290 (9.8)	
Radiation, n (%)			
No	6957 (69.8)	2416 (81.3)	P<0.001
Yes	3003 (30.2)	554 (18.7)	
Chemotherapy, n (%)			
No	5232 (52.5)	1126 (37.9)	P<0.001
Yes	4728 (47.5)	1844 (62.1)	
Tumor size, n (%)			
≤5	6217 (62.4)	1223 (41.2)	P<0.001
>5	3743 (37.6)	1747 (58.8)	
M stage (mean (SD))	0.00 (0.00)	1.00 (0.00)	P<0.001

DM (-), patients without distant metastasis; DM (+), patients with distant metastasis.

**Table 2:** Clinical and pathological characteristics of the study population for the internal validation set.

Variables	DM (-)	DM (+)	P value
	4310	1232	
Age, n (%)			
≤50	422 (9.8)	173 (14.0)	P<0.001
>50	3888 (90.2)	1059 (86.0)	
Sex, n (%)			
Male	1633 (37.9)	391 (31.7)	P<0.001
Female	2677 (62.1)	841 (68.3)	
Race, n (%)			
White	3022 (70.1)	924 (75.0)	P=0.001
Black	544 (12.6)	150 (12.2)	
Other	744 (17.3)	158 (12.8)	
Marital, n (%)			
Married	2553 (59.2)	737 (59.8)	P=0.184
Single	611 (14.2)	194 (15.7)	
Other	1146 (26.6)	301 (24.4)	
Grade, n (%)			
Grade I	609 (14.1)	45 (3.7)	P<0.001
Grade II	1268 (29.4)	309 (25.1)	
Grade III	2320 (53.8)	839 (68.1)	
Grade IV	113 (2.6)	39 (3.2)	
T stage, n (%)			
T1	1154 (26.8)	206 (16.7)	P<0.001
T2	690 (16.0)	84 (6.8)	
T3	1480 (34.3)	256 (20.8)	
T4	791 (18.4)	354 (28.7)	
Tx	195 (4.5)	332 (26.9)	
N stage, n (%)			
N0	2249 (52.2)	389 (31.6)	P<0.001
N1	936 (21.7)	477 (38.7)	
N2	489 (11.3)	104 (8.4)	
N3	530 (12.3)	137 (11.1)	
Nx	106 (2.5)	125 (10.1)	
Radiation, n (%)			
No	2994 (69.5)	1004 (81.5)	P<0.001
Yes	1316 (30.5)	228 (18.5)	
Chemotherapy, n (%)			
No	2262 (52.5)	485 (39.4)	P<0.001
Yes	2048 (47.5)	747 (60.6)	
Tumor size, n (%)			
≤5	2671 (62.0)	493 (40.0)	P<0.001
>5	1639 (38.0)	739 (60.0)	
M stage (mean (SD))	0.00 (0.00)	1.00 (0.00)	P<0.001

DM (-), patients without distant metastasis; DM (+), patients with distant metastasis.

**Table 3:** Clinical and pathological characteristics of the study population for the external validation set.

n	DM (-)	DM (+)	P value
	1,277	318	
Age, n (%)			
≤50	164 (12.8)	52 (16.4)	P=0.122
>50	1113 (87.2)	266 (83.6)	
Sex, n (%)			
Male	572 (44.8)	203 (63.8)	P<0.001
Female	705 (55.2)	115 (36.2)	
Race, n (%)			
White	0 (0)	0 (0)	NA
Black	0 (0)	0 (0)	
Other	1277 (100.0)	318 (100.0)	
Marital, n (%)			
Married	1175 (92.0)	285 (89.6)	P=0.209
Single	102 (8.0)	33 (10.4)	
Other			
Grade, n (%)			
Grade I	147 (11.5)	12 (3.8)	P<0.001
Grade II	463 (36.3)	100 (31.4)	
Grade III	656 (51.4)	198 (62.3)	
Grade IV	11 (0.9)	8 (2.5)	
T stage, n (%)			
T1	235 (18.4)	53 (16.7)	P<0.001
T2	192 (15.0)	18 (5.7)	
T3	537 (42.1)	90 (28.3)	
T4	288 (22.6)	112 (35.2)	
Tx	25 (2.0)	45 (14.2)	
N stage (%)			
N0	257 (20.1)	0 (0.0)	P<0.001
N1	513 (40.2)	20 (6.3)	
N2	266 (20.8)	192 (60.4)	
N3	241 (18.9)	106 (33.3)	
Radiation, n (%)			
No	1274 (99.8)	312 (98.1)	P=0.002
Yes	3 (0.2)	6 (1.9)	
Chemotherapy, n (%)			
No	913 (71.5)	180 (56.6)	P<0.001
Yes	364 (28.5)	138 (43.4)	
Tumor size, n (%)			
≤5	859 (67.3)	133 (41.8)	P<0.001
>5	418 (32.7)	185 (58.2)	
M stage (mean (SD))	0.00 (0.00)	1.00 (0.00)	P<0.001

DM (-), patients without distant metastasis; DM (+), patients with distant metastasis.

### Data collection and processing

SEER patient data were taken from “SEER Research Plus Data, 18 Registries, Nov 2020 Sub (2000-2018)” and extracted using SEER * STAT (8.4.0) software. We processed data from 1595 externally validated patients from the First Hospital of Jilin University according to the standards of SEER data. Data collected for the external validation cohort included age, gender, race, marriage, pathologic grade, tumor size, T stage, N stage, postoperative radiotherapy, and postoperative chemotherapy status. All relevant pathologic indicators were processed according to the 7th edition of the AJCC TNM staging and SEER-related guidelines (**Supplementary Table 1**), while categorical variables were coded (**Supplementary Table 2**).

### Statistical analysis

All data in this study were analyzed and processed using R (version 4.2.3, http://www.r-project.org) and Python (version 3.8, Python Software Foundation, http://www.python.org). The categorization parameters were expressed as numerical values and proportions. The chi-square test was used to compare the between-group differences between the external validation set and the SEER dataset. We used LASSO regression to screen meaningful combinations of features for predicting the risk of distant metastasis in gastric cancer patients. The screened features were analyzed by multifactorial logistic regression analysis to identify independent risk factors for distant metastasis of gastric cancer. The ORs and confidence intervals for each factor were calculated. The identified independent risk factors will be used in constructing machine learning prediction models. We randomly divided the SEER data into a training set and an internal test set in a ratio of 7:3. SVM (Support Vector Machine), MLP (Multilayer Perceptron), XGBoost (Extreme Gradient Boosting), RF (Random Forest), LR (Logistic Regression) and KNN (K Nearest Neighbors) were performed in the training set to develop predictive models. The relative importance ranking of each input variable is analyzed in each model. We used 10-fold cross-validation in the training set to evaluate the discriminant ability of the model and each independent predictor using ROC curves. We assess the model by using the area under the curve (AUC). The closer the AUC is to 1.0, the higher the authenticity of the detection method. When the AUC equals 0.5, the realism is the lowest, and there is no application value. Although the ROC-AUC approach contains a lot of helpful information for evaluation, it is not a one-size-fits-all measure, so when the dataset is unbalanced, we validate the AUC values by plotting the precision-recall curves and calculating the AUPRC, which takes into account the performance of the model under different Precision and Recall values. For AUPRC, larger values are better, with values close to 1 indicating that the model performs very well in the classification task. Decision curve analysis is a method for evaluating predictive models, and we also plotted decision curves to compare the predictive validity of the models, defining the model with the best performance based on the maximum AUC in internal and external validation. For model interpretation, we used SHAP analysis to determine the contribution of each feature in the sample to prediction. Blue indicates features that hurt disease prediction, leading to a decrease in SHAP value; red indicates features that positively affect disease prediction, leading to an increase in SHAP value. Finally, we developed a network calculator for clinical use based on the predictive model that has been constructed. The code for the article data analysis is represented in the supporting material (**Supplementary Table 3**).

## Result

### Baseline population characteristics

This study included 18,472 patients with gastric cancer from the SEER database. Among them, 4202 (22.75%) had distant metastases, and 14270 (77.25%) did not have distant metastases. The demographic and clinicopathologic characteristics of all these patients are shown in **Tables 1**, **2**. The patients in the SEER database were randomized in a 7:3 ratio into the training set (n = 12930) and the internal test set (n = 5542). The external validation used data from 1595 gastric cancer patients in the First Hospital of Jilin University (**Table 3**). Details of the training set, internal test set and external validation set are shown in **Tables 1**–**3**.

We analyzed the differences between patients in the metastatic and non-metastatic groups in the SEER database and came to some of the following conclusions. We included 10 clinicopathological factors of relevance in this study: age, gender, marital status, race, differentiation grade, tumor size, T-stage, N-stage, radiotherapy, and chemotherapy. Patients in the training set of the SEER database were divided into a DM(-) subgroup (9,960 patients without distant metastases, 77.0%) and a DM(+) subgroup (2,970 patients with distant metastases, 23.0%). In our study, the proportion of younger patients was higher in the DM(+) subgroup compared with DM(-) (P < 0.01). In the DM(+) subgroup, the distant metastasis rate was much higher in women than in men (P=0.01). And outside, the distant metastasis rate was higher in unmarried (1715/7518, 22.8%) than in married (488/1868, 26.1%, P=0.01). In comparing the progression of gastric cancer in the two subgroups, the proportion of patients with tumors larger than 5 cm in size was higher in the DM(+) subgroup (58.8%) than in the DM(-) subgroup (37.6%; P<0.001). There were more unclear T-stages in the DM(+) subgroup (P < 0.001) and more lymph node metastases in this group (P < 0.001). There was a significant difference in access to treatment between patients in the DM(+) and DM(-) subgroups (P < 0.001).

### Univariate and multiple logistic regression analysis

**Figure 2:**
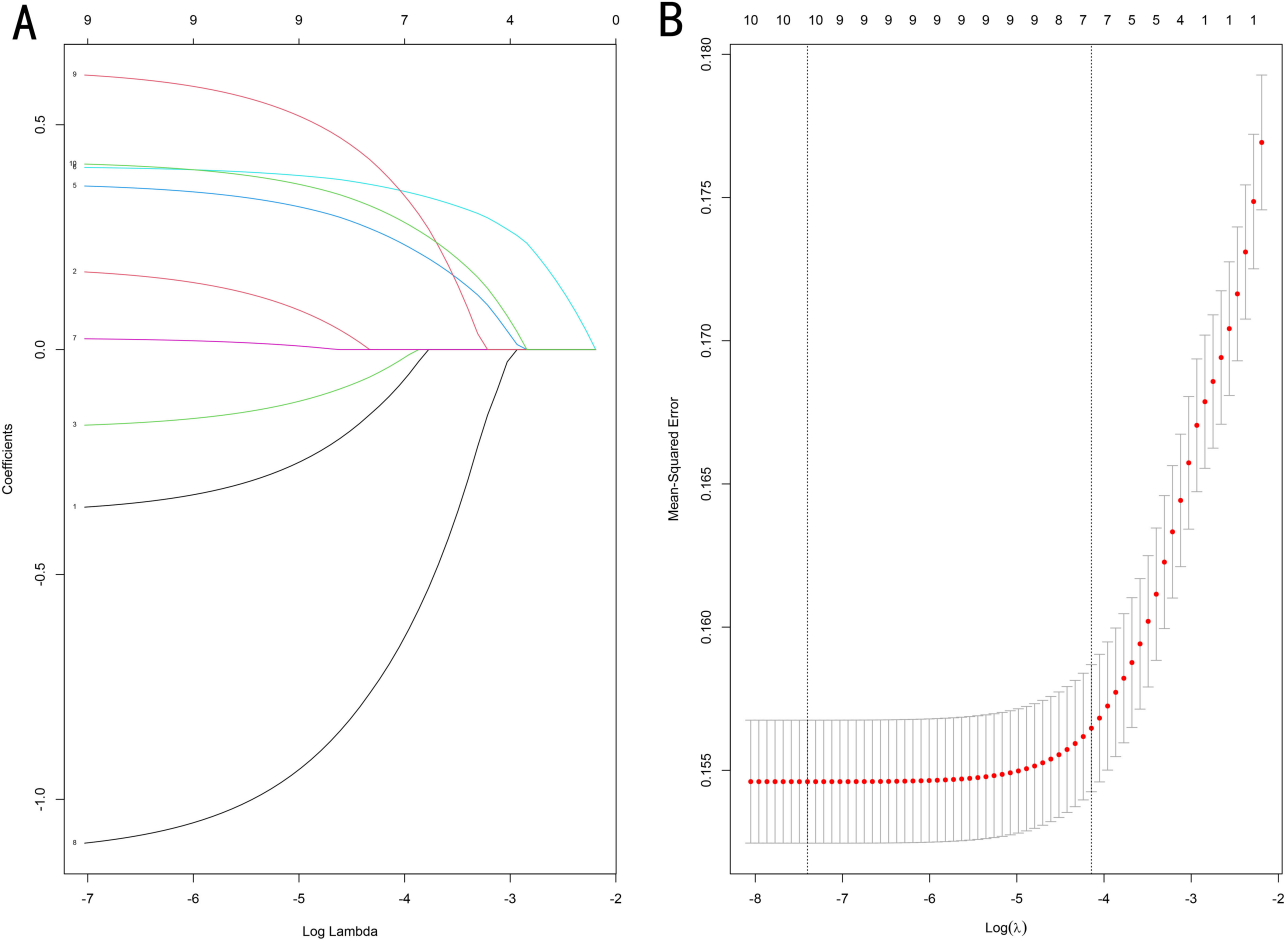
**(A)** Optimal parameter (λ) selection in the LASSO model, with the optimal tuning parameter log(λ) in the horizontal coordinate and the regression coefficients in the vertical coordinate. **(B)** Distribution of LASSO coefficients for the clinical factors, with the optimal tuning parameter log(λ) in the horizontal coordinate and the binomial deviation in the vertical coordinate.

We performed LASSO regression and multivariate logistic regression analyses on the training set data to identify the variables incorporated into the machine learning model. Based on the LASSO regression analysis results, we screened seven meaningful combinations of features, including age, race, differentiation grading, tumor size, T stage, radiotherapy and chemotherapy (**Figure 2**). Incorporating the above features into the multivariate logistic regression analysis showed that age, race, differentiation grade, tumor size, T stage, radiotherapy and chemotherapy were independent risk factors for distant metastasis of gastric cancer (P<0.05, **Table 4**). We included variables with P<0.05 in the multiple logistic regression analysis for machine learning analysis.

**Table 4:** Univariate and multiple logistic regression analysis of variables in SEER database.

Dependent: M stage	Category	DM (-) (N=14270)	DM (+) (N=4202)	Multiple Analysis	
		n (%)	n (%)	Odds Ratio (95% CI)	P value
Age	≤50	1357 (9.5%)	601 (14.3)	Ref	Ref
	>50	12913 (90.5%)	3601 (85.7%)	0.71 (0.63-0.80)	P<0.001
Race	White	9784 (68.6%)	3060 (72.8%)	Ref	Ref
	Black	1812 (12.7%)	524 (12.5%)	0.91 (0.81-1.02)	P=0.120
	Other	2674 (18.7%)	618 (14.7%)	0.68 (0.61-0.75)	P<0.001
Grade	Grade I	2011 (14.1%)	137 (3.3%)	Ref	Ref
	Grade II	4180 (29.3%)	1005 (23.9%)	3.12 (2.56-3.80)	P<0.001
	Grade III	7696 (53.9%)	2907 (69.2%)	4.13 (3.41-5.00)	P<0.001
	Grade IV	383 (2.7%)	153 (3.6%)	3.92 (2.97-5.17)	P<0.001
T stage	T1	3934 (27.6%)	745 (17.7%)	Ref	Ref
	T2	2299 (16.1%)	275 (6.5%)	0.51 (0.44-0.60)	P<0.001
	T3	4815 (33.7%)	879 (20.9%)	0.56 (0.50-0.63)	P<0.001
	T4	2571 (18%)	1226 (29.2%)	1.23 (1.09-1.38)	P<0.001
	Tx	651 (4.6%)	1077 (25.6%)	6.02 (5.27-6.88)	P<0.001
Radiation	No	9951 (69.7%)	3420 (81.4%)	Ref	Ref
	Yes	4319 (30.3%)	782 (18.6%)	0.36 (0.33-0.40)	P<0.001
Chemotherapy	No	7494 (52.5%)	1611 (38.3%)	Ref	Ref
	Yes	6776 (47.5%)	2591 (61.7%)	2.37 (2.17-2.58)	P<0.001
Tumor size	≤5	8888 (62.3%)	1716 (40.8%)	Ref	Ref
	>5	5382 (37.7%)	2486 (59.2%)	1.92 (1.77-2.09)	P<0.001

CI, confidence interval; Ref, reference.

### Model performance

**Figure 3:**
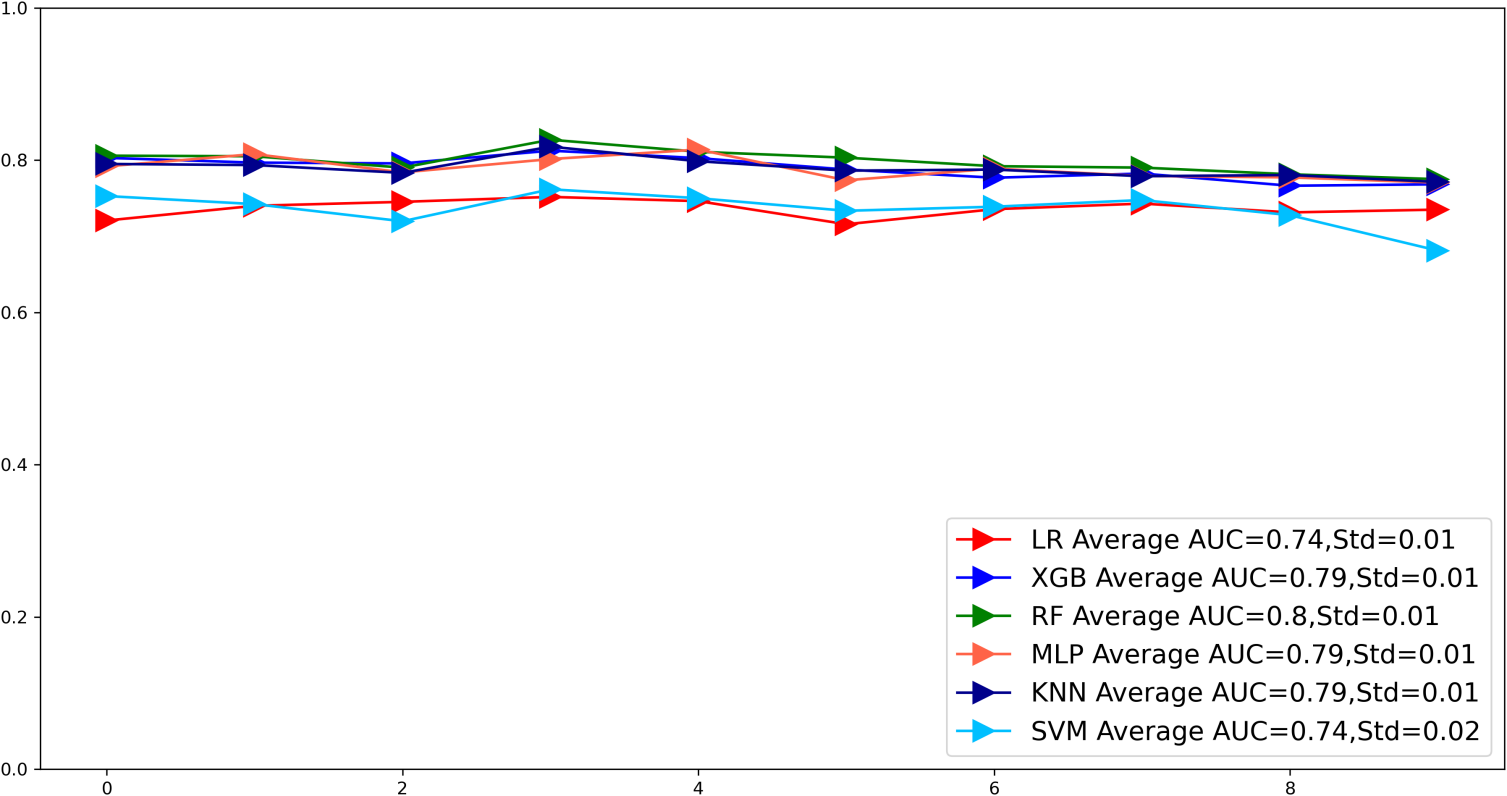
The plot of 10-fold cross-validation. LR, Logistic regression; MLP, Multilayer perceptron; XGB, The extreme gradient boosting machine; RF, Random Forest; SVM, Support vector machine; KNN, K-nearest neighbor; AUC, area under the curve.

**Figure 4:**
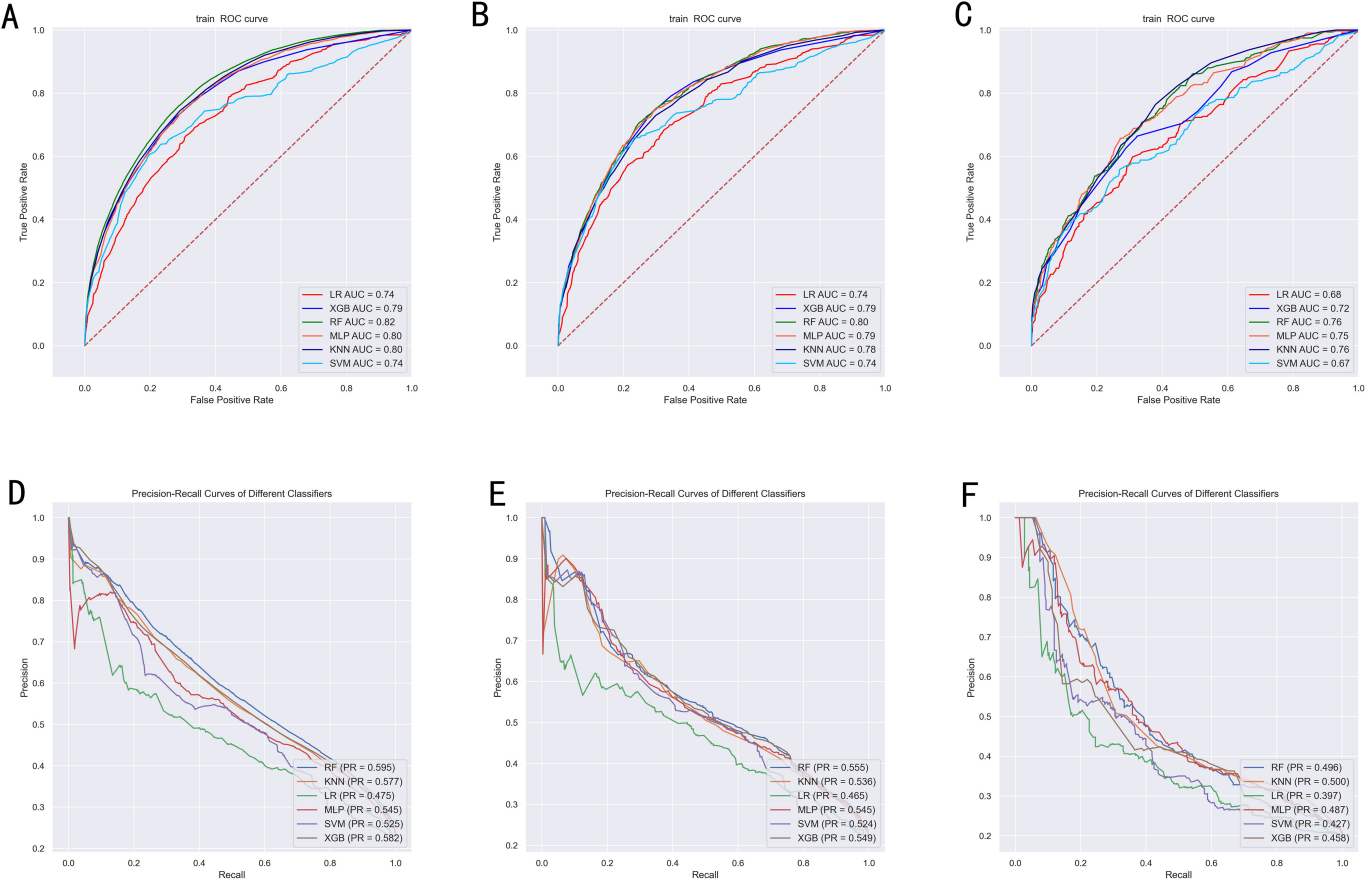
**(A)** ROC curves of eight machine learning models in the training set. **(B)** ROC curves of eight machine learning models in the internal validation set. **(C)** ROC curves of eight machine learning models in the external validation set. **(D)** PR curves of eight machine learning models in the training set. **(E)** PR curves of eight machine learning models in the internal validation set. **(F)** PR curves of eight machine learning models in the external validation set. PR, Precision-recall; ROC, Receiver operating characteristic curve.

**Figure 5:**
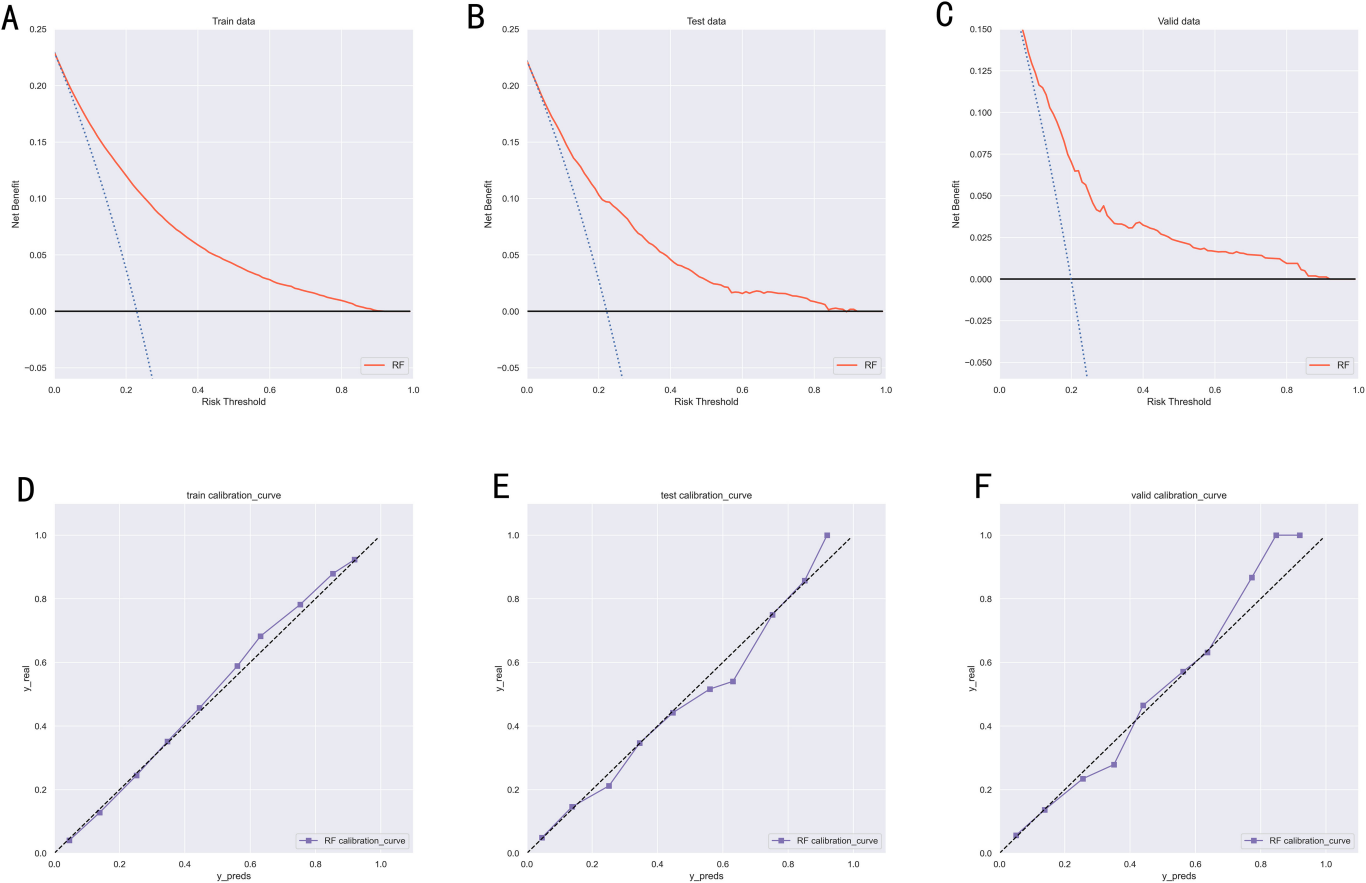
**(A)** DCA curves of RF model in the training set. **(B)** DCA curves of RF model in the internal validation set. **(C)** DCA curves of RF model in the external validation set. **(D)** Calibration curves of RF model in the training set. **(E)** Calibration curves of RF model in the internal validation set. **(F)** Calibration curves of RF model in the external validation set. DCA, Decision curve analysis; RF, Random Forest.

**Figure 6:**
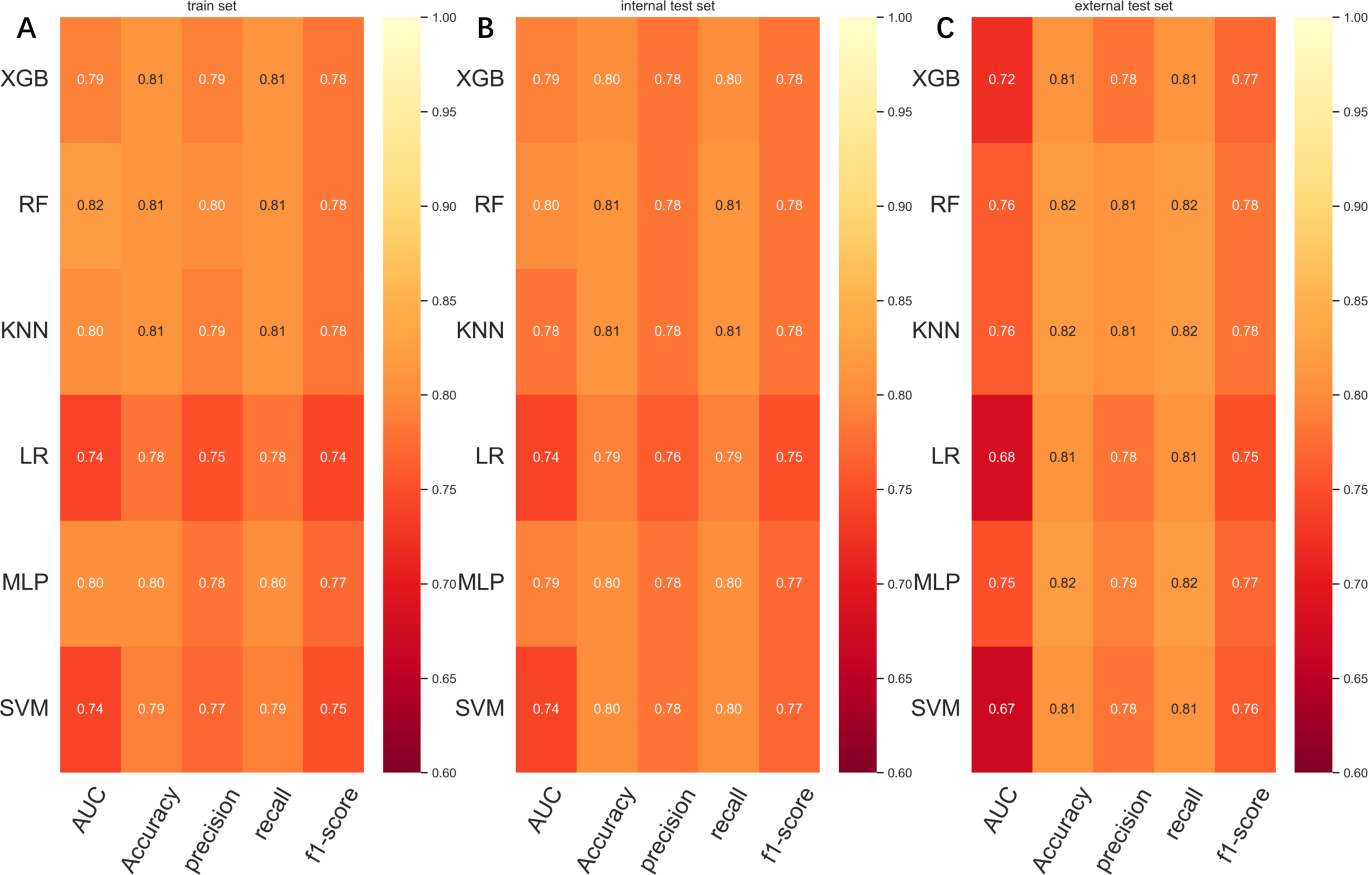
**(A)** Prediction performance of six models in the training set. **(B)** Prediction performance of six models in the internal test set. **(C)** Prediction performance of six models in the external validation set. AUC, Area under the curve; LR, Logistic regression; KNN, K-nearest neighbor; RF, Random Forest; XGB, The extreme gradient boosting machine; MLP, Multilayer perceptron; SVM, Support vector machine.

To compare the prediction performance of the six models, we performed ten-fold cross-validation on the training set data (**Figure 3**). The average AUC values of the six machine learning models ranged between 0.74 and 0.80, showing excellent predictive ability. The RF algorithm had the highest average AUC value (AUC=0.8, SD=0.01). **Figure 4** shows the PR curves for each model in the training set, with the RF model having a higher PR value than the other five models (AUPRC=0.595) (**Figure 4D**). RF also showed strong predictive ability in the clinical decision curve analysis (**Figure 5A**). **Figure 5D** shows the calibration curve of the RF model in the training set, indicating that the RF model has a more accurate prediction performance. In conclusion, the RF model has high reliability. **Figures 4**, **5** also show the ROC curves, PR curves, calibration curves, and DCA curves of RF for both the internal test set and external validation set for the six models. The RF model performs well in both datasets, showing discriminative ability beyond other models. The heat map analysis results in a comprehensive, clear, intuitive, and easy-to-judge analysis method that is suitable for a comprehensive assessment because it provides a clearer picture of the model’s performance in multiple dimensions (**Figure 6**). After a thorough review of the model performance in the three datasets, we concluded that the RF model performed best in predicting distant metastasis in gastric cancer patients and designated the RF model as the best model.

### The relative importance of variables in machine learning algorithms

**Figure 7:**
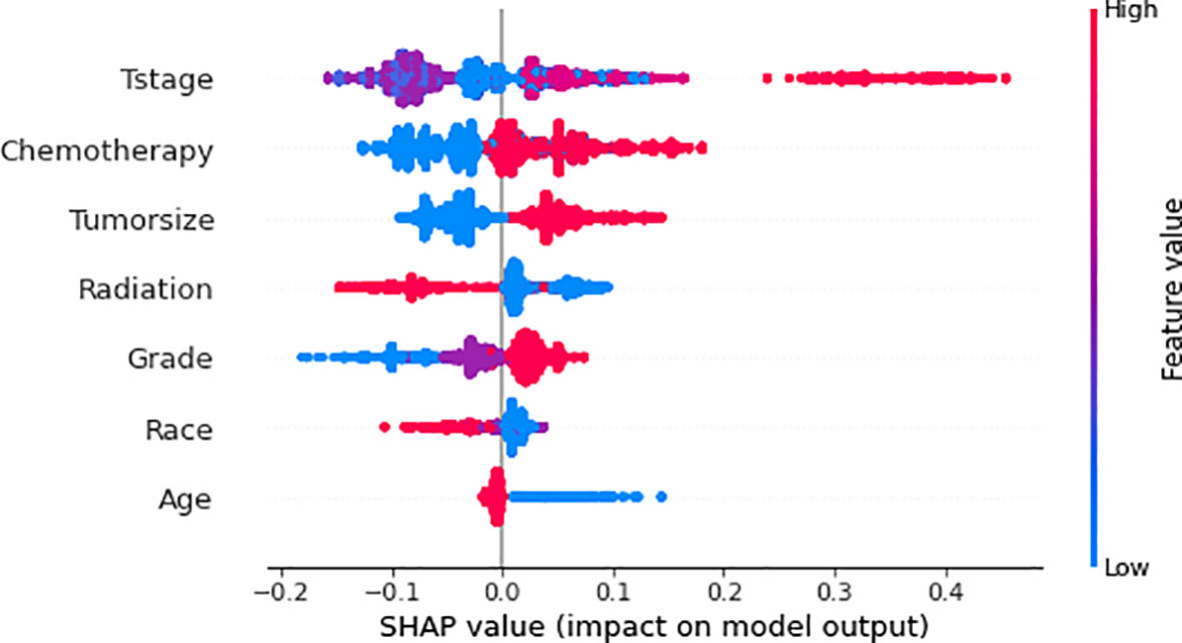
Relative importance of variables based on SHAP for XGB prediction model. SHAP, Shapley’s Additive explanations; RF, Random Forest.

We use SHAP to interpret RF model. Generally, the higher the SHAP value of a feature, the higher the probability of the target event occurring. In SHAP analysis, red indicates feature values that positively affect the model, and blue indicates feature values that negatively affect the model (18). The results of the SHAP analysis showed that the T-stage of the tumor was the most critical variable, followed by chemotherapy, tumor size, radiotherapy, differentiation grading, race, and age (**Figure 7**).

### Web calculator

**Figure 8:**
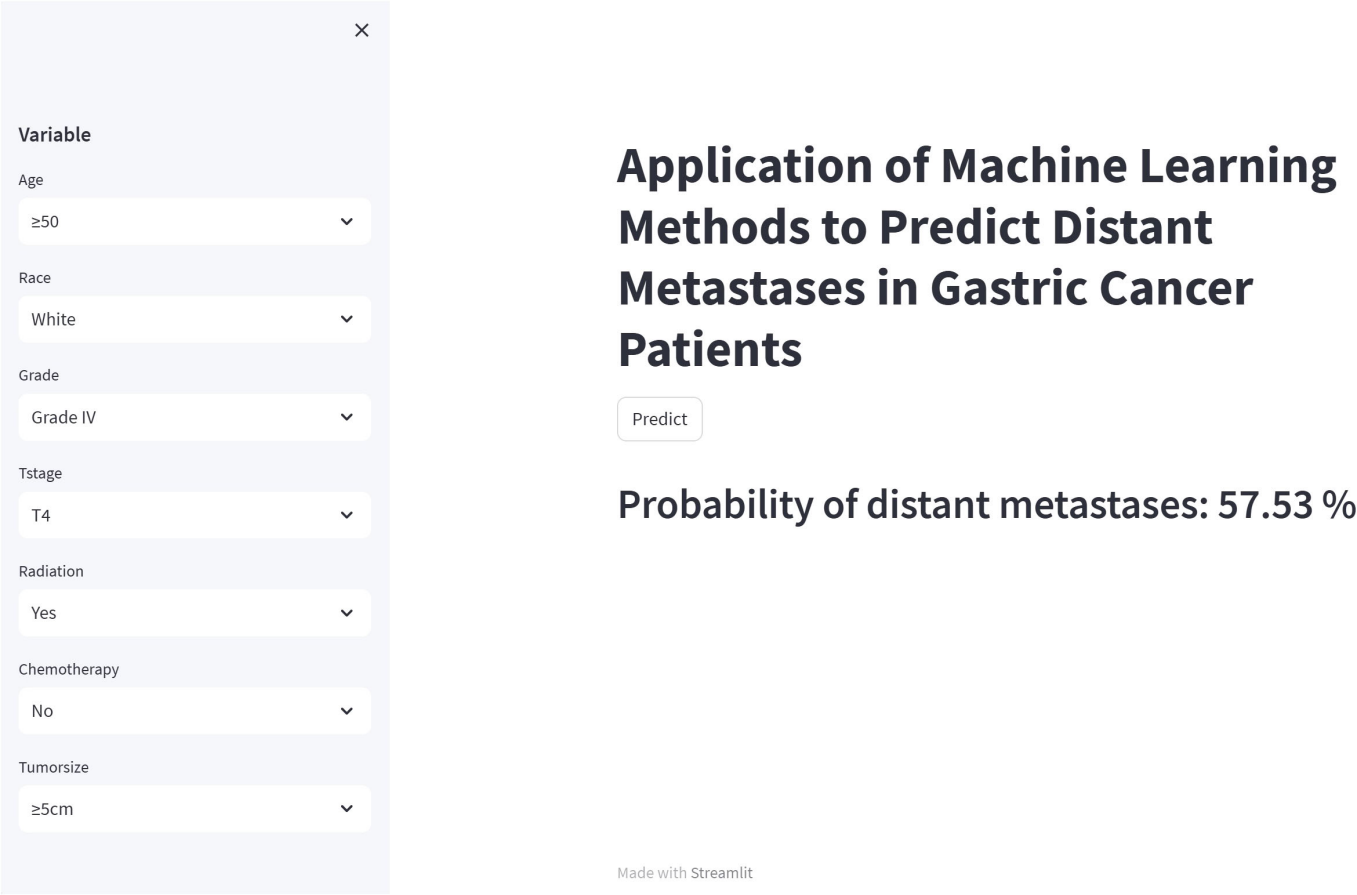
A web calculator for predicting distant metastasis from gastric cancer.

Although the RF model is the best performing of the six machine learning models we used, it is complex and unsuitable for clinical generalization. Therefore, we built a network calculator based on the RF model, and the probability of distant metastasis in this gastric cancer patient can be derived by inputting the relevant clinicopathologic information of the patient into the built network calculator. The image of the network calculator is shown in **Figure 8**. The link to the network calculator is https://share.streamlit.io/woshiwz/gastric_cancer/main/gastricMet.py.

## Discussion

Gastric cancer is one of the more prevalent malignant tumors of the gastrointestinal tract, often presenting with distant metastases. It is the third leading cause of cancer-related deaths in men and the fifth in women (19). The low survival rate is primarily attributed to delayed diagnosis (20). At the time of diagnosis, 35% of gastric cancer patients have developed distant metastases, 31% have peritoneal metastases, 14% have liver metastases, and 16% have lung metastases (21). Peritoneal metastasis and liver metastasis are the two most common sites of metastasis in gastric cancer, so routine abdominal CT is often recommended for gastric cancer patients. Positron emission tomography/computed tomography (PET/CT) is also an effective diagnostic method for distant metastases. Still, it is not routinely used as a screening modality for distant metastases due to its more expensive cost and higher radiation exposure (22–24). Based on the limitations of routine screening, this study constructed a prediction model based on clinical and pathological indicators to predict the probability of distant metastasis in patients with gastric cancer, thus identifying patients at risk of developing distant metastasis.

To date, numerous research experts have worked on constructing diverse models for predicting distal metastasis of gastric cancer carcinomas. For example, a study by Dong et al. developed a radiological column line diagram based on CT phenotype and Lauren type for predicting peritoneal metastasis of gastric cancer (25). To the best of our knowledge, this paper is the first to use machine learning algorithms and to build a network calculator that can be used to predict distal metastasis of gastric cancer based on an optimal model. Our study found that RF is the best algorithm to predict distant metastasis of gastric cancer among the six machine learning algorithms. RF algorithm is a machine learning algorithm with multiple special decision trees (26). It has the advantage of having a strong predictive power that prevents overfitting (27).

We used descriptive statistics and logistic regression to analyze the variables associated with distant metastasis of gastric cancer. Additionally, we utilized SHAP values to evaluate the impact of each factor on the prediction model. Our analysis of the SHAP values revealed that each variable contributed to the model (**Figure 5**). According to George and Keklikoglou et al., chemotherapy may increase the probability of distant metastasis in malignant tumors, which may be because chemotherapy promotes the expression of metastatic genes, as well as increasing the secretion of exosomes that can contribute to the increase in metastasis (28, 29). Exosomes are small membrane-encapsulated vesicles 30-200 nm in diameter, enriched with specific proteins, lipids, mRNAs and miRNAs (30). The role of exosomal RNA in gastric cancer metastasis involves all steps, including EMT, cancer cell invasion, organ metastasis, and pre-metastatic ecological niche formation (31). These studies have shown that chemotherapy not only makes the malignant tumors in the body shrink but also increases the probability of distant metastasis of the tumors. Radiotherapy is now being used with increasing frequency as adjuvant therapy for gastrointestinal malignancies. The findings of the INT0116 trial show the critical role of postoperative radiotherapy in the adjuvant treatment of gastric cancer (32). Our study found that radiotherapy can reduce the probability of distant metastasis of gastric cancer, which may be because postoperative radiotherapy can remove cancer cells that may remain after surgery and reduce the possibility of cancer cells growing again at the primary site. Some studies have shown that radiotherapy can reduce the risk of distant metastasis of gastric cancer by targeting the irradiation of the lymph node area and reducing the number of cancer cells in the lymph nodes. However, a study by Tepper also showed that radiotherapy has a greater tendency to distant metastasis in gastric cancer (33), so more research is needed to investigate whether radiotherapy can promote distant metastasis of gastric cancer. In our study, tumor size was another important risk factor for the development of distant metastasis in gastric cancer. This is consistent with the findings of Kooby’s team (34). Zhou’s team showed that gastric cancer patients with larger tumors had a higher risk of invasive growth, lymph node metastasis, and distant metastasis characterized by peritoneal dissemination. As a result, the prognosis of these patients was poorer. Their study also showed that patients with larger tumors had a higher risk of lymph node metastasis and were more prone to distant metastasis, thus affecting prognosis (35). We found that younger patients with gastric cancer were more likely to develop distant metastases. A previous study showed that GC in younger patients (40 years old or younger) tended to exhibit more aggressive tumor behavior compared to older patients (36). Thus, younger patients may have a greater probability of developing distant metastases. Our study also found that the T-stage is an independent risk factor for the development of distant metastasis in gastric cancer. This may be because the deeper the tumor’s infiltration depth would result in the lower expression of a protein called MPC1. Zhou et al. found that MPC1 overexpression significantly impaired the migratory and invasive abilities of GC cells and inhibited the proliferation, migration and invasion of GC cells (37). The results of a study conducted by Zhang and his team showed that white patients were more likely to have metastatic gastric cancer than Asian patients (38), which may be related to routine screening practices in Asia (39). Screening can significantly reduce the incidence and mortality of gastric cancer and achieve early diagnosis and early treatment of gastric cancer. However, population-based screening is not routinely recommended in the United States, which results in more patients being found to have progressed to an advanced stage and, therefore, a worse prognosis for white patients (40–42).

We constructed a new prediction model based on independent predictors to accurately predict the risk of distant metastasis in gastric cancer. By comparing the AUC values of the models constructed by various machine learning algorithms, we chose an optimal prediction model, and the calibration curve showed that the model had sound predictive effects, suggesting that the model is expected to provide important reference information for tumor surveillance and clinical decision-making. Moreover, we used data from the First Hospital of Jilin University in China for external independent validation to improve the credibility of the results of this study. Finally, we developed a network calculator based on the constructed optimal model. Users can obtain predictive results regarding the risk of developing distant metastases after gastric cancer surgery by simply selecting options from those provided based on the patient’s relevant clinical and pathologic parameters. The development of this calculator can give guidance value to clinicians in making clinical decisions.

Although our model has some advantages, there are still some shortcomings that need to be further optimized. First, this study is retrospective, and there may be some biases, such as selectivity, information, and other data biases. Second, the external validation data applied in this study all came from one medical center, and these may ultimately affect the construction of the predictive model, so more external validation data from more medical centers are needed to validate our predictive model. Third, due to the data limitation of the SEER database, we have some essential variables, such as blood biochemical indexes, nerve and vascular infiltration, and other information cannot be obtained in time, thus limiting the further optimization of our model.

## Conclusion

We constructed six models that can predict distant metastasis of gastric cancer based on six machine learning algorithms. Among them, the RF model showed the most robust predictive performance in the internal test set and the external validation set, with a strong predictive ability. Therefore, we hope that the prediction model constructed by RF model can help clinicians identify patients with a high risk of distant metastasis of gastric cancer earlier and provide timely intervention and treatment.

## Data Availability

The original contributions presented in the study are included in the article/**Supplementary Material**. Further inquiries can be directed to the corresponding author.
