# Supplementary material for: Applying machine learning techniques to predict the risk of distant metastasis from gastric cancer: a real world retrospective study
Source: Front Oncol. 2024 Dec 5;14:1455914. doi: 10.3389/fonc.2024.1455914 (PMC11655338; doi:10.3389/fonc.2024.1455914)
Supplement: Supplementary file 1 [file Table1.docx]

**Supplement Table 1. Normalization standards of clinical data in outer validation set.**

| **Clinical data** | **Normalization Standard** |
| --- | --- |
| **Basic demographic data** |  |
| Age | Age at diagnosis. |
| Marital status | Marital status at diagnosis |
| Sex | Sex |
| Race | Race |
| **Tumor information** |  |
| Tumor size | the largest diameter of the primary tumor. |
| TNM stage | According to AJCC 7th edition. |
| Tumor grade | Well differentiated, Moderately differentiated, Poorly differentiated, and Undifferentiated. |

**Abbreviation:** AJCC, American Joint Committee on Cancer;
