# Supplementary material for: Applying machine learning techniques to predict the risk of distant metastasis from gastric cancer: a real world retrospective study
Source: Front Oncol. 2024 Dec 5;14:1455914. doi: 10.3389/fonc.2024.1455914 (PMC11655338; doi:10.3389/fonc.2024.1455914)
Supplement: Supplementary file 2 [file Table2.docx]

**Supplement Table 2.** References for property values of clinical features in models.

| **Variables** | **Property Values** | |
| --- | --- | --- |
| **Age**  ≤50  >50  **Sex** | | **0**  **1** |
| Male | | **0** |
| Female | | **1** |
| **Race** | |  |
| White | | **0** |
| Black | | **1** |
| Other | | **2** |
| **T stage** | |  |
| T1 | | **0** |
| T2 | | **1** |
| T3 | | **2** |
| T4  Tx | | **3**  **4** |
| **N stage** | |  |
| N0 | | **0** |
| N1 | | **1** |
| N2  N3  Nx | | **2**  **3**  **4** |
| **Grade** | |  |
| Well differentiated; Grade I | | **0** |
| Moderately differentiated; Grade II | | **1** |
| Poorly differentiated; Grade III | | **2** |
| Undifferentiated; anaplastic; Grade IV | | **3** |
| **Marital Status** | |  |
| Married (including common law) | | **0** |
| Unmarried | | **1** |
| Other  **Tumor size**  ≤5cm  ＞5cm  **Chemotherapy**  No  Yes  **Radiation**  No  Yes | | **2**  **0**  **1**  **0**  **1**  **0**  **1** |
