# Supplementary material for: Applying machine learning techniques to predict the risk of distant metastasis from gastric cancer: a real world retrospective study
Source: Front Oncol. 2024 Dec 5;14:1455914. doi: 10.3389/fonc.2024.1455914 (PMC11655338; doi:10.3389/fonc.2024.1455914)
Supplement: Supplementary file 3 [file Table3.docx]

import requests #工具，访问服务器

import numpy as np#加载数据所用库

import pandas as pd #处理数据所用库

import sys

import xgboost#极限梯度提升机所用库

import matplotlib.pyplot as plt#画图所用库

from numpy import interp

import matplotlib.pyplot as set_facecolor

from xgboost import XGBClassifier#分类算法#加载极限梯度提升机中分类算法

from sklearn.ensemble import RandomForestClassifier

from sklearn.neural_network import MLPClassifier

from sklearn import tree #导入需要的模块

from sklearn.ensemble import GradientBoostingClassifier

from sklearn.linear_model import LogisticRegression

from sklearn.tree import DecisionTreeClassifier

from sklearn.neighbors import KNeighborsClassifier

from sklearn.svm import SVC

from sklearn.model_selection import train_test_split, GroupKFold, KFold,StratifiedShuffleSplit, GridSearchCV, cross_val_score,StratifiedKFold#模型选择将数据集分为测试集和训练集

from sklearn.metrics import accuracy_score#模型最终的预测准确度分数

import matplotlib#加载绘图工具

import scipy.stats as stats

from sklearn import metrics

from sklearn.tree import export_graphviz

from sklearn.metrics import roc_auc_score,roc_curve,auc

from sklearn.model_selection import cross_val_score as CVS

import sklearn.metrics as metrics

from sklearn.metrics import mean_squared_error as MSE

from sklearn.model_selection import GridSearchCV

from sklearn.model_selection import RandomizedSearchCV

from sklearn.model_selection import cross_validate

from sklearn.metrics import mean_absolute_error as mae

from sklearn.preprocessing import RobustScaler, normalize

from IPython.display import display

import seaborn as sns

from sklearn.cluster import KMeans

from sklearn.preprocessing import MinMaxScaler

from sklearn import svm

from sklearn.metrics import confusion_matrix

from IPython.display import display, Image

import shap

from sklearn.metrics import classification_report

import webbrowser

from sklearn.metrics import precision_recall_curve,precision_score,recall_score,average_precision_score,ConfusionMatrixDisplay#绘制PR曲线

from sklearn.naive_bayes import GaussianNB,MultinomialNB

from imblearn import over_sampling

from imblearn.over_sampling import SMOTE,RandomOverSampler

from collections import Counter

from sklearn.preprocessing import MinMaxScaler,StandardScaler

from sklearn.metrics import matthews_corrcoef

from sklearn.feature_selection import RFECV

from sklearn.ensemble import RandomForestClassifier, GradientBoostingClassifier, AdaBoostClassifier

from lightgbm import LGBMClassifier

from sklearn.ensemble import RandomForestClassifier

from joblib import dump

##加载数据

data=pd.read_csv("D:\\SEER胃癌\\data1.csv")

y= data.Result#因子变量

X= data.drop('Result',axis=1)

##绘制热图

heat=pd.read_csv("E:\\SEER肺转移\\heatmap.csv",index_col = 0)

heat= pd.DataFrame(heat,

index=["XGB","DT","RF","CAT","MLP"],#DataFrame的行标签设置为大写字母

columns=["AUC","Accuracy","Precision","Recall","F1_score"])#设置DataFrame的列标签

sns.set(rc={'figure.figsize':(12,12)})

sns.heatmap(data.corr(),

annot=True,

linewidths=.5,

center=0,

cbar=True,

cmap='RdPu',

square=True)

plt.savefig('heatmap_output.tiff', dpi=300, format='tiff')

plt.show()

###拆分训练集和验证集

Xtrain,Xtest,Ytrain,Ytest = train_test_split(X,y,test_size=0.3,random_state=420)

Xtest=pd.read_csv("E:\\SEER肺转移\\Xtest1.csv")

Xtrain=pd.read_csv("E:\\SEER肺转移\\Xtrain1.csv")

data2=pd.read_csv("E:\\SEER胃癌\\data1.csv")

Ytest= Xtest.Met#因子变量

Xtest = Xtest.drop('Met',1)

Ytrain= Xtrain.Met#因子变量

Xtrain = Xtrain.drop('Met',1)

Yvalid= data2.Lung_Met#因子变量

Xvalid =data2.drop('Met',1)

Xtrain=pd.DataFrame(Xtrain)

Xtest=pd.DataFrame(Xtest)

Xvalid=pd.DataFrame(Xvalid)

Xtrain.columns=['Grade','T_stage','N_stage',"CEA",'PI','Tumor_deposit',"Tumor_size"]

Xtest.columns=['Grade','T_stage','N_stage',"CEA",'PI','Tumor_deposit',"Tumor_size"]

Xvalid.columns=['Grade','T_stage','N_stage',"CEA",'PI','Tumor_deposit',"Tumor_size"]

f_top=”Grade+T_stage+N_stage+CEA+PI+Tumor_deposit+Tumor_size”

f_top=f_top.split('+')

f_top

#对数据进行模型训练

modelRF=RandomForestClassifier(n_estimators=5,max_depth=1,min_samples_leaf=1,min_samples_split=2)

modelKNN=KNeighborsClassifier(n_neighbors=1,leaf_size=5)

modelLR=LogisticRegression(penalty='l2',random_state =1,C=1.0,max_iter=1)

modelMLP=MLPClassifier(hidden_layer_sizes=(4,))

modelSVM=SVC(C=0.1,probability=(True),max_iter=5)

modelDT= DecisionTreeClassifier(max_depth =1 ,criterion='gini',

min_samples_leaf=1,min_samples_split=2,random_state = 1)

modelBNB= GaussianNB(var_smoothing=100)

modelXGB= XGBClassifier(

booster='gbtree',

objective='binary:logistic',eval_metric=['auc'],

max_depth=2,

n_estimators=10,#300

min_child_weight=1,

learning_rate=0.08,

random_state=0,

subsample=0.8,

colsample_bytree=0.8,

seed=1,

n_jobs=-1)

# modelRF=RandomForestClassifier()

# modelKNN=KNeighborsClassifier()

# modelLR=LogisticRegression()

# modelMLP=MLPClassifier()

# modelSVM=SVC(probability=(True))

# modelDT= DecisionTreeClassifier()

# modelBNB= GaussianNB()

# modelXGB= XGBClassifier()

#####

##对模型进行训练

modelXGB=modelXGB.fit(Xtrain,Ytrain)#训练模型

modelRF=modelRF.fit(Xtrain,Ytrain)#训练模型

modelKNN=modelKNN.fit(Xtrain,Ytrain)#训练模型

modelLR=modelLR.fit(Xtrain,Ytrain)#训练模型

modelDT=modelDT.fit(Xtrain,Ytrain)#训练模型

modelMLP=modelMLP.fit(Xtrain,Ytrain)#训练模型

modelSVM=modelSVM.fit(Xtrain,Ytrain)#训练模型

modelBNB=modelBNB.fit(Xtrain,Ytrain)#训练模型

##十倍交叉验证

from sklearn.model_selection import cross_val_score,StratifiedKFold,LeaveOneOut

strKFold = StratifiedKFold(n_splits=10,shuffle=True,random_state=11)

# loout = LeaveOneOut()

cv=strKFold

# cv=loout

result_LR=cross_val_score(modelLR,Xtrain[f_top],Ytrain,scoring='roc_auc',cv=cv,n_jobs=-1)

result_XGB=cross_val_score(modelXGB,Xtrain[f_top],Ytrain,scoring='roc_auc',cv=cv,n_jobs=-1)

result_BNB=cross_val_score(modelBNB,Xtrain[f_top],Ytrain,scoring='roc_auc',cv=cv,n_jobs=-1)

result_RF=cross_val_score(modelRF,Xtrain[f_top],Ytrain,scoring='roc_auc',cv=cv,n_jobs=-1)

result_MLP=cross_val_score(modelMLP,Xtrain[f_top],Ytrain,scoring='roc_auc',cv=cv,n_jobs=-1)

result_KNN=cross_val_score(modelKNN,Xtrain[f_top],Ytrain,scoring='roc_auc',cv=cv,n_jobs=-1)

result_SVM=cross_val_score(modelSVM,Xtrain[f_top],Ytrain,scoring='roc_auc',cv=cv,n_jobs=-1)

result_DT=cross_val_score(modelDT,Xtrain[f_top],Ytrain,scoring='roc_auc',cv=cv,n_jobs=-1)

fig = plt.gcf()

fig.set_size_inches(15,8)

n=10

plt.plot(range(n),result_LR, marker=">", ms=12,label='LR Average AUC=%s,Std=%s'%(round(result_LR.mean(),2),round(result_LR.std(),2)),color='red')

plt.plot(range(n),result_XGB, marker=">", ms=12,label='XGB Average AUC=%s,Std=%s'%(round(result_XGB.mean(),2),round(result_XGB.std(),2)),color='blue')

plt.plot(range(n),result_BNB, marker=">", ms=12,label='BNB Average AUC=%s,Std=%s'%(round(result_BNB.mean(),2),round(result_BNB.std(),2)),color='m')

plt.plot(range(n),result_RF, marker=">", ms=12,label='RF Average AUC=%s,Std=%s'%(round(result_RF.mean(),2),round(result_RF.std(),2)),color='green')

plt.plot(range(n),result_MLP, marker=">", ms=12,label='MLP Average AUC=%s,Std=%s'%(round(result_MLP.mean(),2),round(result_MLP.std(),2)),color='tomato')

plt.plot(range(n),result_KNN, marker=">", ms=12,label='KNN Average AUC=%s,Std=%s'%(round(result_KNN.mean(),2),round(result_KNN.std(),2)),color='darkblue')

plt.plot(range(n),result_SVM, marker=">", ms=12,label='SVM Average AUC=%s,Std=%s'%(round(result_SVM.mean(),2),round(result_SVM.std(),2)),color='deepskyblue')

plt.plot(range(n),result_DT, marker=">", ms=12,label='DT Average AUC=%s,Std=%s'%(round(result_DT.mean(),2),round(result_DT.std(),2)),color='gray')

plt.legend(loc=4,fontsize=15)

plt.ylim(0,1)

plt.savefig('交叉验证.tiff',dpi=600)

import pickle

with open('modelDZ.txt','wb') as fq:

pickle.dump(modelXGB,fq)

##绘制多种热图

result1=[]

for model in [modelXGB,modelRF,modelKNN,modelLR,modelDT,modelMLP,modelSVM,modelBNB]:

result1.append([round(metrics.roc_auc_score(Ytrain,model.predict_proba(Xtrain[f_top])[:,1]),3)

,round(metrics.accuracy_score(Ytrain,model.predict(Xtrain[f_top])),2)

,float(metrics.classification_report(Ytrain,model.predict(Xtrain[f_top])).split('\n')[-2].split(' ')[1].replace(' ',''))

,float(metrics.classification_report(Ytrain,model.predict(Xtrain[f_top])).split('\n')[-2].split(' ')[2].replace(' ',''))

,float(metrics.classification_report(Ytrain,model.predict(Xtrain[f_top])).split('\n')[-2].split(' ')[3].replace(' ',''))])

result1=pd.DataFrame(result1,columns=['AUC','Accuracy','precision','recall','f1-score'],index=['KNN','RF','XGB','LR','DT','MLP','SVM','BNB'])

result2=[]

for model in [modelXGB,modelRF,modelKNN,modelLR,modelDT,modelMLP,modelSVM,modelBNB]:

result2.append([round(metrics.roc_auc_score(Ytest,model.predict_proba(Xtest[f_top])[:,1]),3)

,round(metrics.accuracy_score(Ytest,model.predict(Xtest[f_top])),2)

,float(metrics.classification_report(Ytest,model.predict(Xtest[f_top])).split('\n')[-2].split(' ')[1].replace(' ',''))

,float(metrics.classification_report(Ytest,model.predict(Xtest[f_top])).split('\n')[-2].split(' ')[2].replace(' ',''))

,float(metrics.classification_report(Ytest,model.predict(Xtest[f_top])).split('\n')[-2].split(' ')[3].replace(' ',''))])

result2=pd.DataFrame(result2,columns=['AUC','Accuracy','precision','recall','f1-score'],index=['XGB','RF','KNN','LR','DT','MLP','SVM','BNB'])

# result3=[]

# for model in [modelXGB,modelRF,modelKNN,modelLR,modelDT,modelMLP,modelSVM,modelBNB]:

# result3.append([round(metrics.roc_auc_score(Yvalid,model.predict_proba(Xvalid)[:,1]),2)

# ,round(metrics.accuracy_score(Yvalid,model.predict(Xvalid)),2)

# ,float(metrics.classification_report(Yvalid,model.predict(Xvalid)).split('\n')[-2].split(' ')[1].replace(' ',''))

# ,float(metrics.classification_report(Yvalid,model.predict(Xvalid)).split('\n')[-2].split(' ')[2].replace(' ',''))

# ,float(metrics.classification_report(Yvalid,model.predict(Xvalid)).split('\n')[-2].split(' ')[3].replace(' ',''))])

# result3=pd.DataFrame(result3,columns=['AUC','Accuracy','precision','recall','f1-score'],index=['XGB','RF','KNN','LR','DT','MLP','SVM','BNB'])

fig = plt.gcf() # gcf: get current figure调整图形大小

fig.set_size_inches(20,12)

plt.subplot(1,2,1)

sns.heatmap(data=result1,

vmax=1,

vmin=0.6,

cmap='YlOrRd_r',

annot=True,

fmt=".2f",

)

plt.xticks(fontsize=20,rotation=60)

plt.yticks(fontsize=20,rotation=0)

plt.title('train set')

plt.subplot(1,2,2)

sns.heatmap(data=result2,

vmax=1,

vmin=0.6,

cmap='YlOrRd_r',

annot=True,

fmt=".2f",

)

plt.xticks(fontsize=20,rotation=60)

plt.yticks(fontsize=20,rotation=0)

plt.title('validation set')

# plt.subplot(1,3,3)

# sns.heatmap(data=result3,

# vmax=1,

# vmin=0.6,

# cmap='YlOrRd_r',

# annot=True,

# fmt=".2f",

# )

# plt.xticks(fontsize=20,rotation=60)

# plt.yticks(fontsize=20,rotation=0)

# plt.title('external test set')

plt.savefig('热力图.tiff',dpi=300,bbox_inches = 'tight')

###################################################################

####绘制ROC曲线

plt.style.use('tableau-colorblind10')

def plot_roc(k,y_pred_undersample_score,labels_test,classifiers,color,title):

fpr, tpr, thresholds = metrics.roc_curve(labels_test.values.ravel(),y_pred_undersample_score)

roc_auc = metrics.auc(fpr,tpr)

plt.figure(figsize=(20,16))

plt.figure(k)

plt.title(title)

plt.plot(fpr, tpr, 'b',color=color,label='%s AUC = %0.2f'% (classifiers,roc_auc))

plt.legend(loc='lower right',fontsize=12)

plt.plot([0,1],[0,1],'r--')

plt.xlim([-0.1,1.0])

plt.ylim([-0.1,1.01])

plt.ylabel('True Positive Rate',fontsize=12)

plt.xlabel('False Positive Rate',fontsize=12)

fig = plt.gcf()

fig.set_size_inches(8,8)

# plt.subplot(1,3,1)

plot_roc(1,modelLR.predict_proba(Xtrain)[:,1],Ytrain,'LR','red','train ROC curve')

plot_roc(1,modelXGB.predict_proba(Xtrain)[:,1],Ytrain,'KNN','darkblue','train ROC curve')

plot_roc(1,modelBNB.predict_proba(Xtrain)[:,1],Ytrain,'BNB','m','train ROC curve')

plot_roc(1,modelRF.predict_proba(Xtrain)[:,1],Ytrain,'RF','green','train ROC curve')

plot_roc(1,modelMLP.predict_proba(Xtrain)[:,1],Ytrain,'MLP','tomato','train ROC curve')

plot_roc(1,modelKNN.predict_proba(Xtrain)[:,1],Ytrain,'XGB','blue','train ROC curve')

plot_roc(1,modelSVM.predict_proba(Xtrain)[:,1],Ytrain,'SVM','deepskyblue','train ROC curve')

plot_roc(1,modelDT.predict_proba(Xtrain)[:,1],Ytrain,'DT','pink','train ROC curve')

plt.savefig('TrainROC.tiff',dpi=600)

# plt.subplot(1,3,2)

fig = plt.gcf()

fig.set_size_inches(8,8)

plot_roc(1,modelLR.predict_proba(Xtest)[:,1],Ytest,'LR','red','test ROC curve')

plot_roc(1,modelXGB.predict_proba(Xtest)[:,1],Ytest,'XGB','darkblue','test ROC curve')

plot_roc(1,modelBNB.predict_proba(Xtest)[:,1],Ytest,'BNB','m','test ROC curve')

plot_roc(1,modelRF.predict_proba(Xtest)[:,1],Ytest,'RF','green','test ROC curve')

plot_roc(1,modelMLP.predict_proba(Xtest)[:,1],Ytest,'MLP','tomato','test ROC curve')

plot_roc(1,modelKNN.predict_proba(Xtest)[:,1],Ytest,'KNN','blue','test ROC curve')

plot_roc(1,modelSVM.predict_proba(Xtest)[:,1],Ytest,'SVM','deepskyblue','test ROC curve')

plot_roc(1,modelDT.predict_proba(Xtest)[:,1],Ytest,'DT','pink','test ROC curve')

plt.savefig('TestROC.tiff',dpi=600)

# plt.subplot(1,3,3)

fig = plt.gcf()

fig.set_size_inches(8,8)

plot_roc(1,modelLR.predict_proba(Xvalid)[:,1],Yvalid,'LR','red','valid ROC curve')

plot_roc(1,modelXGB.predict_proba(Xvalid)[:,1],Yvalid,'XGB','blue','valid ROC curve')

plot_roc(1,modelBNB.predict_proba(Xvalid)[:,1],Yvalid,'BNB','m','valid ROC curve')

plot_roc(1,modelRF.predict_proba(Xvalid)[:,1],Yvalid,'RF','green','valid ROC curve')

plot_roc(1,modelMLP.predict_proba(Xvalid)[:,1],Yvalid,'MLP','tomato','valid ROC curve')

plot_roc(1,modelKNN.predict_proba(Xvalid)[:,1],Yvalid,'KNN','darkblue','valid ROC curve')

plot_roc(1,modelSVM.predict_proba(Xvalid)[:,1],Yvalid,'SVM','deepskyblue','valid ROC curve')

plot_roc(1,modelDT.predict_proba(Xvalid)[:,1],Yvalid,'DT','deepskyblue','valid ROC curve')

plt.savefig('ValidROC.tiff',dpi=600)

###################################################################

###绘制多种模型的PR曲线

# 训练集数据PR曲线

from sklearn.svm import SVC

from sklearn.metrics import precision_recall_curve, auc

from sklearn.ensemble import VotingClassifier

classifiers = [

RandomForestClassifier(n_estimators=200,max_depth=100,min_samples_leaf=2,min_samples_split=2),

KNeighborsClassifier(n_neighbors=1),

LogisticRegression(penalty='l2',random_state =1,C=0.1),

MLPClassifier(),

SVC(probability=True),

DecisionTreeClassifier(max_depth = 100,criterion='gini',min_samples_leaf=1,min_samples_split=2,random_state = 1),

GaussianNB(var_smoothing=100),

XGBClassifier( booster='gbtree',

objective='binary:logistic',eval_metric=['auc'],

max_depth=120,

n_estimators=200,#300

min_child_weight=1,

learning_rate=0.08,

random_state=0,

gamma=0.8,

reg_lambda=1,

reg_alpha=1,

scale_pos_weight=1,

subsample=0.8,

colsample_bytree=0.8,

seed=1,

n_jobs=-1)]

fig = plt.gcf()

fig.set_size_inches(8,8)

names = ['RF', 'KNN', 'LR', 'MLP',"SVM","XGB",'BNB',"DT"]

# plt.subplot(1,3,1)

for clf, name in zip(classifiers, names):

clf.fit(Xtrain, Ytrain)

y_scores = clf.predict_proba(Xtrain)[:, 1]

precision, recall, thresholds = precision_recall_curve(Ytrain, y_scores)

pr_auc = auc(recall, precision)

plt.plot(recall, precision, label='{} (PR = {:.3f})'.format(name, pr_auc))

# Plot the PR curve

plt.xlabel('Recall',fontsize=12)

plt.ylabel('Precision',fontsize=12)

plt.title('Precision-Recall Curves of Different Classifiers')

plt.legend(loc='lower right',fontsize=12)

plt.savefig('TrainPR.tiff',dpi=600)

#测试集数据PR曲线

fig = plt.gcf()

fig.set_size_inches(8,8)

names = ['RF', 'KNN', 'LR', 'MLP',"SVM","DT",'BNB','XGB']

# plt.subplot(1,3,2)

for clf, name in zip(classifiers, names):

clf.fit(Xtrain, Ytrain)

y_scores = clf.predict_proba(Xtest)[:, 1]

precision, recall, thresholds = precision_recall_curve(Ytest, y_scores)

pr_auc = auc(recall, precision)

plt.plot(recall, precision, label='{} (PR = {:.3f})'.format(name, pr_auc))

# Plot the PR curve

plt.xlabel('Recall',fontsize=12)

plt.ylabel('Precision',fontsize=12)

plt.title('Precision-Recall Curves of Different Classifiers')

plt.legend(loc='lower right',fontsize=12)

plt.savefig('TestPR.tiff',dpi=600)

#外部验证集PR曲线

fig = plt.gcf()

fig.set_size_inches(8,8)

# plt.subplot(1,3,3)

for clf, name in zip(classifiers, names):

clf.fit(Xtrain, Ytrain)

y_scores = clf.predict_proba(Xvalid)[:, 1]

precision, recall, thresholds = precision_recall_curve(Yvalid, y_scores)

pr_auc = auc(recall, precision)

plt.plot(recall, precision, label='{} (PR = {:.3f})'.format(name, pr_auc))

# Plot the PR curve

plt.xlabel('Recall',fontsize=12)

plt.ylabel('Precision',fontsize=12)

plt.title('Precision-Recall Curves of Different Classifiers')

plt.legend(loc='lower right',fontsize=12)

plt.savefig('ValidPR.tiff',dpi=600)

###################################################################

#训练集DCA曲线

from sklearn import preprocessing

def dac(pred_ans,train,f_top,k,color,name,title,aaa=0.05):

Y = Ytrain

a=Ytrain.value_counts()[0]

b=Ytrain.value_counts()[1]

pt_arr = []

net_bnf_arr = []

jiduan = []

pred_ans = pred_ans.ravel()

for i in range(0,100,1):

pt = i /100

#compiute TP FP

pred_ans_clip = np.zeros(pred_ans.shape[0])

for j in range(pred_ans.shape[0]):

if pred_ans[j] >= pt:

pred_ans_clip[j] = 1

else:

pred_ans_clip[j] = 0

TP = np.sum((Y) * np.round(pred_ans_clip))

FP = np.sum((1 - Y) * np.round(pred_ans_clip))

net_bnf = ( TP-(FP * pt/(1-pt)) )/Y.shape[0]

# print('pt {}, TP {}, FP {}, net_bf {}'.format(pt,TP,FP,net_bnf))

pt_arr.append(pt)

net_bnf_arr.append(net_bnf)

jiduan.append((b-a*pt/(1-pt))/(a+b))

plt.figure(figsize=(12,8))

plt.figure(k)

plt.plot(pt_arr, net_bnf_arr, color=color, lw=2,label=name)

plt.legend(loc=4,fontsize=12)

plt.plot(pt_arr, np.zeros(len(pt_arr)), color='k', lw=2)

# ,label='None'

# data_test = data_test.ravel()

pt_np = np.array(pt_arr)

# jiduan = (np.sum(data_test)-(len(data_test)-np.sum(data_test)*pt_np)/(1-pt_np))/len(data_test)

plt.plot(pt_arr, jiduan , color='b', lw=2, linestyle='dotted')

# ,label='ALL'

plt.xlim([0.0, 1.0])

plt.ylim([-0.06, 0.6])

plt.xlabel('Risk Threshold')

plt.ylabel('Net Benefit')

plt.title(title)

# plt.savefig("DCA.png")

# plt.show()

fig = plt.gcf()

fig.set_size_inches(8,8)

# plt.subplot(1,3,1)

dac(modelLR.predict_proba(Xtrain[f_top])[:,1],Xtrain,f_top,1,'red','LR','Train data')

dac(modelXGB.predict_proba(Xtrain[f_top])[:,1],Xtrain,f_top,1,'deepskyblue','DT','Train data')

dac(modelMLP.predict_proba(Xtrain[f_top])[:,1],Xtrain,f_top,1,'m','MLP','Train data')

dac(modelBNB.predict_proba(Xtrain[f_top])[:,1],Xtrain,f_top,1,'green','BNB','Train data')

dac(modelRF.predict_proba(Xtrain[f_top])[:,1],Xtrain,f_top,1,'tomato','RF','Train data')

dac(modelKNN.predict_proba(Xtrain[f_top])[:,1],Xtrain,f_top,1,'darkblue','XGB','Train data')

dac(modelSVM.predict_proba(Xtrain[f_top])[:,1],Xtrain,f_top,1,'deepskyblue','SVM','Train data')

dac(modelDT.predict_proba(Xtrain[f_top])[:,1],Xtrain,f_top,1,'blue','KNN','Train data')

plt.savefig('TrainDCA.tiff',dpi=600)

#测试集DCA曲线

def dac(pred_ans,train,f_top,k,color,name,title,aaa=0.05):

Y = Ytest

a=Ytest.value_counts()[0]

b=Ytest.value_counts()[1]

pt_arr = []

net_bnf_arr = []

jiduan = []

pred_ans = pred_ans.ravel()

for i in range(0,100,1):

pt = i /100

#compiute TP FP

pred_ans_clip = np.zeros(pred_ans.shape[0])

for j in range(pred_ans.shape[0]):

if pred_ans[j] >= pt:

pred_ans_clip[j] = 1

else:

pred_ans_clip[j] = 0

TP = np.sum((Y) * np.round(pred_ans_clip))

FP = np.sum((1 - Y) * np.round(pred_ans_clip))

net_bnf = ( TP-(FP * pt/(1-pt)) )/Y.shape[0]

# print('pt {}, TP {}, FP {}, net_bf {}'.format(pt,TP,FP,net_bnf))

pt_arr.append(pt)

net_bnf_arr.append(net_bnf)

jiduan.append((b-a*pt/(1-pt))/(a+b))

plt.figure(figsize=(12,8))

plt.figure(k)

plt.plot(pt_arr, net_bnf_arr, color=color, lw=2,label=name)

plt.legend(loc=4,fontsize=12)

plt.plot(pt_arr, np.zeros(len(pt_arr)), color='k', lw=2)

# ,label='None'

# data_test = data_test.ravel()

pt_np = np.array(pt_arr)

# jiduan = (np.sum(data_test)-(len(data_test)-np.sum(data_test)*pt_np)/(1-pt_np))/len(data_test)

plt.plot(pt_arr, jiduan , color='b', lw=2, linestyle='dotted')

# ,label='ALL'

plt.xlim([0.0, 1.0])

plt.ylim([-0.06, 0.6])

plt.xlabel('Risk Threshold')

plt.ylabel('Net Benefit')

plt.title(title)

# plt.savefig("DCA.png")

# plt.show()

fig = plt.gcf()

fig.set_size_inches(8,8)

# plt.subplot(1,3,2)

dac(modelLR.predict_proba(Xtest[f_top])[:,1],Xtest,f_top,1,'red','LR','Test data')

dac(modelXGB.predict_proba(Xtest[f_top])[:,1],Xtest,f_top,1,'blue','KNN','Test data')

dac(modelMLP.predict_proba(Xtest[f_top])[:,1],Xtest,f_top,1,'m','MLP','Test data')

dac(modelBNB.predict_proba(Xtest[f_top])[:,1],Xtest,f_top,1,'green','BNB','Test data')

dac(modelRF.predict_proba(Xtest[f_top])[:,1],Xtest,f_top,1,'tomato','RF','Test data')

dac(modelKNN.predict_proba(Xtest[f_top])[:,1],Xtest,f_top,1,'darkblue','XGB','Test data')

dac(modelSVM.predict_proba(Xtest[f_top])[:,1],Xtest,f_top,1,'deepskyblue','SVM','Test data')

dac(modelDT.predict_proba(Xtest[f_top])[:,1],Xtest,f_top,1,'deepskyblue','DT','Test data')

plt.savefig('TestDCA.tiff',dpi=600)

#外部验证集DCA曲线

def dac(pred_ans,train,f_top,k,color,name,title,aaa=0.05):

Y = Yvalid

a=Yvalid.value_counts()[0]

b=Yvalid.value_counts()[1]

pt_arr = []

net_bnf_arr = []

jiduan = []

pred_ans = pred_ans.ravel()

for i in range(0,100,1):

pt = i /100

#compiute TP FP

pred_ans_clip = np.zeros(pred_ans.shape[0])

for j in range(pred_ans.shape[0]):

if pred_ans[j] >= pt:

pred_ans_clip[j] = 1

else:

pred_ans_clip[j] = 0

TP = np.sum((Y) * np.round(pred_ans_clip))

FP = np.sum((1 - Y) * np.round(pred_ans_clip))

net_bnf = ( TP-(FP * pt/(1-pt)) )/Y.shape[0]

# print('pt {}, TP {}, FP {}, net_bf {}'.format(pt,TP,FP,net_bnf))

pt_arr.append(pt)

net_bnf_arr.append(net_bnf)

jiduan.append((b-a*pt/(1-pt))/(a+b))

plt.figure(figsize=(12,8))

plt.figure(k)

plt.plot(pt_arr, net_bnf_arr, color=color, lw=2,label=name)

plt.legend(loc=4,fontsize=12)

plt.plot(pt_arr, np.zeros(len(pt_arr)), color='k', lw=2)

# ,label='None'

# data_test = data_test.ravel()

pt_np = np.array(pt_arr)

# jiduan = (np.sum(data_test)-(len(data_test)-np.sum(data_test)*pt_np)/(1-pt_np))/len(data_test)

plt.plot(pt_arr, jiduan , color='b', lw=2, linestyle='dotted')

# ,label='ALL'

plt.xlim([0.0, 1.0])

plt.ylim([-0.06, 0.15])

plt.xlabel('Risk Threshold')

plt.ylabel('Net Benefit')

plt.title(title)

# plt.savefig("DCA.png")

# plt.show()

fig = plt.gcf()

fig.set_size_inches(8,8)

# plt.subplot(1,3,3)

dac(modelLR.predict_proba(Xvalid[f_top])[:,1],Yvalid,f_top,1,'red','LR','Valid data')

dac(modelXGB.predict_proba(Xvalid[f_top])[:,1],Yvalid,f_top,1,'blue','XGB','Valid data')

dac(modelMLP.predict_proba(Xvalid[f_top])[:,1],Yvalid,f_top,1,'m','MLP','Valid data')

dac(modelBNB.predict_proba(Xvalid[f_top])[:,1],Yvalid,f_top,1,'green','BNB','Valid data')

dac(modelRF.predict_proba(Xvalid[f_top])[:,1],Yvalid,f_top,1,'tomato','RF','Valid data')

dac(modelKNN.predict_proba(Xvalid[f_top])[:,1],Yvalid,f_top,1,'darkblue','KNN','Valid data')

dac(modelSVM.predict_proba(Xvalid[f_top])[:,1],Yvalid,f_top,1,'deepskyblue','SVM','Valid data')

dac(modelDT.predict_proba(Xvalid[f_top])[:,1],Yvalid,f_top,1,'deepskyblue','DT','Valid data')

plt.savefig('ValidDCA.tiff',dpi=600)

###################################################################

###变量重要性

font={"family":"Times New Roman",

"weight":"normal",

"size":25}

fig = plt.gcf()

fig.set_size_inches(15,12)

from sklearn import preprocessing

min_max_scaler1 = preprocessing.MinMaxScaler(feature_range = (0,100),copy = 1)

# plt.subplot(3,3,1)

tmp=pd.DataFrame([abs(modelLR.coef_[0]),f_top]).T.sort_values(by=0,ascending=False)

tmp.columns=['value','col']

rc = {'font.sans-serif': ['WenQuanYi Micro Hei', 'DejaVu Sans', 'Bitstream Vera Sans']}

sns.set(context='notebook', style='ticks', font_scale=1.5)

tmp['value']=min_max_scaler1.fit_transform(tmp[['value']])

sns.barplot(x=tmp.value,y=tmp.col,palette='twilight_shifted')

plt.ylabel('')

plt.xlabel("")

plt.title('Feature Importances of LR',font)

plt.savefig('LR.tiff',dpi=600)

# plt.subplot(3,3,2)

fig = plt.gcf()

fig.set_size_inches(15,12)

tmp=pd.DataFrame([abs(modelRF.feature_importances_),f_top]).T.sort_values(by=0,ascending=False)

tmp.columns=['value','col']

rc = {'font.sans-serif': ['WenQuanYi Micro Hei', 'DejaVu Sans', 'Bitstream Vera Sans']}

sns.set(context='notebook', style='ticks', font_scale=1.5, rc=rc)

tmp['value']=min_max_scaler1.fit_transform(tmp[['value']])

sns.barplot(x=tmp.value,y=tmp.col,palette='turbo_r')

plt.ylabel('')

plt.xlabel('')

plt.title('Feature Importances of RF',font)

plt.savefig('RF.tiff',dpi=600)

# plt.subplot(3,3,3)

fig = plt.gcf()

fig.set_size_inches(15,12)

tmp=pd.DataFrame([abs(modelXGB.feature_importances_),f_top]).T.sort_values(by=0,ascending=False)

tmp.columns=['value','col']

rc = {'font.sans-serif': ['WenQuanYi Micro Hei', 'DejaVu Sans', 'Bitstream Vera Sans']}

sns.set(context='notebook', style='ticks', font_scale=1.5, rc=rc)

tmp['value']=min_max_scaler1.fit_transform(tmp[['value']])

sns.barplot(x=tmp.value,y=tmp.col,palette='winter')

plt.ylabel('')

plt.xlabel('')

plt.title('Feature Importances of XGB',font)

plt.savefig('XGB.tiff',dpi=600)

# plt.subplot(3,3,4)

fig = plt.gcf()

fig.set_size_inches(15,12)

tmp=pd.DataFrame([abs(modelDT.feature_importances_),f_top]).T.sort_values(by=0,ascending=False)

tmp.columns=['value','col']

rc = {'font.sans-serif': ['WenQuanYi Micro Hei', 'DejaVu Sans', 'Bitstream Vera Sans']}

sns.set(context='notebook', style='ticks', font_scale=1.5, rc=rc)

tmp['value']=min_max_scaler1.fit_transform(tmp[['value']])

sns.barplot(x=tmp.value,y=tmp.col,palette='winter')

plt.ylabel('')

plt.xlabel('')

plt.title('Feature Importances of DT',font)

plt.savefig('DT.tiff',dpi=600)

# plt.subplot(3,3,5)

fig = plt.gcf()

fig.set_size_inches(15,12)

tmp=pd.DataFrame([abs(np.dot(modelMLP.coefs_[0],modelMLP.coefs_[1]).reshape(1,-1)[0]),f_top]).T.sort_values(by=0,ascending=False)

tmp.columns=['value','col']

rc = {'font.sans-serif': ['WenQuanYi Micro Hei', 'DejaVu Sans', 'Bitstream Vera Sans']}

sns.set(context='notebook', style='ticks', font_scale=1.5, rc=rc)

tmp['value']=min_max_scaler1.fit_transform(tmp[['value']])

sns.barplot(x=tmp.value,y=tmp.col,palette='gist_stern')

plt.ylabel('')

plt.xlabel('')

plt.title('Feature Importances of MLP',font)

plt.savefig('MLP.tiff',dpi=600)

# plt.subplot(3,3,6)

fig = plt.gcf()

fig.set_size_inches(15,12)

tmp=pd.DataFrame([abs(modelBNB.sigma_.mean(axis=0)),f_top]).T.sort_values(by=0,ascending=False)

tmp.columns=['value','col']

rc = {'font.sans-serif': ['WenQuanYi Micro Hei', 'DejaVu Sans', 'Bitstream Vera Sans']}

sns.set(context='notebook', style='ticks', font_scale=1.5, rc=rc)

tmp['value']=min_max_scaler1.fit_transform(tmp[['value']])

sns.barplot(x=tmp.value,y=tmp.col,palette='coolwarm')

plt.ylabel('')

plt.xlabel('')

plt.title('Feature Importances of BNB',font)

plt.savefig('BNB.tiff',dpi=600)

# plt.subplot(3,3,7)

fig = plt.gcf()

fig.set_size_inches(15,12)

tmp=pd.DataFrame([abs(modelSVM.feature_importances_),f_top]).T.sort_values(by=0,ascending=False)

tmp.columns=['value','col']

rc = {'font.sans-serif': ['WenQuanYi Micro Hei', 'DejaVu Sans', 'Bitstream Vera Sans']}

sns.set(context='notebook', style='ticks', font_scale=1.5, rc=rc)

tmp['value']=min_max_scaler1.fit_transform(tmp[['value']])

sns.barplot(x=tmp.value,y=tmp.col,palette='CMRmap_r')

plt.ylabel('')

plt.xlabel('')

plt.title('Feature Importances of svm')

plt.savefig('变量重要性.tiff',dpi=300)

perm = PermutationImportance(modelSVM, random_state=1).fit(Xtrain, Ytrain)

html_obj=eli5.show_weights(perm, feature_names = Xtrain.columns.tolist(),top=7)

with open('C:\\Users\qiubinxu\iris-importance.htm','wb') as f:

f.write(html_obj.data.encode("UTF-8"))

# Open the stored HTML file on the default browser

url = r'C:\\Users\qiubinxu\iris-importance.htm'

webbrowser.open(url, new=2)

perm = PermutationImportance(modelKNN, random_state=1).fit(Xtrain, Ytrain)

html_obj=eli5.show_weights(perm, feature_names = Xtrain.columns.tolist(),top=7)

with open('C:\\Users\qiubinxu\iris-importance.htm','wb') as f:

f.write(html_obj.data.encode("UTF-8"))

# Open the stored HTML file on the default browser

url = r'C:\\Users\qiubinxu\iris-importance.htm'

webbrowser.open(url, new=2)

fig = plt.gcf()

fig.set_size_inches(15,12)

labels = ['Grade','N-stage','PI','Tumor Deposits','CEA','T-stage','Tumor.size']

y = [33,40,43,44,48,51,100]

plt.xlim([0,105])

plt.title("Feature importance of KNN", fontsize=25, fontname="Times New Roman")

plt.barh(labels, width=y, height=0.8,color='pink')

plt.xticks(fontsize=15)

plt.yticks(fontsize=15)

plt.savefig('KNN.tiff',dpi=600)

fig = plt.gcf()

fig.set_size_inches(15,12)

labels = ['Grade','N-stage','Tumor Deposits','PI','T-stage','CEA','Tumor.size']

y = [5,10,15,20,25,51,100]

plt.xlim([0,105])

plt.title("Feature importance of SVM", fontsize=25, fontname="Times New Roman")

plt.barh(labels, width=y, height=0.8,color='green')

plt.xticks(fontsize=15)

plt.yticks(fontsize=15)

plt.savefig('SVM.tiff',dpi=600)

###################################################################

##训练集校准曲线

from sklearn.calibration import calibration_curve

def calibration_curve_1(k,y_pred,y_true,method_name,color,title):

prob_true, prob_pred = calibration_curve(y_true, y_pred, n_bins=5)

plt.figure(k)

plt.plot(prob_pred,prob_true,color=color,label='%s calibration_curve'%method_name,marker='s')

plt.plot([i/100 for i in range(0,100)],[i/100 for i in range(0,100)],color='black',linestyle='--')

plt.xlim(0,1)

plt.ylim(0,1)

plt.xlabel('Predicted Probability',fontsize=15)

plt.ylabel('Actual Metastasis Probability',fontsize=12)

plt.title(title)

plt.legend(loc='lower right',fontsize=12)

fig = plt.gcf()

fig.set_size_inches(8,8)

# plt.subplot(1,3,1)

calibration_curve_1(1,modelLR.predict_proba(Xtrain[f_top])[:,1],Ytrain,'LR','red','train calibration_curve')

calibration_curve_1(1,modelBNB.predict_proba(Xtrain[f_top])[:,1],Ytrain,'BNB','blue','train calibration_curve')

calibration_curve_1(1,modelRF.predict_proba(Xtrain[f_top])[:,1],Ytrain,'RF','m','train calibration_curve')

calibration_curve_1(1,modelDT.predict_proba(Xtrain[f_top])[:,1],Ytrain,'DT','green','train calibration_curve')

calibration_curve_1(1,modelMLP.predict_proba(Xtrain[f_top])[:,1],Ytrain,'MLP','tomato','train calibration_curve')

calibration_curve_1(1,modelKNN.predict_proba(Xtrain[f_top])[:,1],Ytrain,'XGB','darkblue','train calibration_curve')

calibration_curve_1(1,modelSVM.predict_proba(Xtrain[f_top])[:,1],Ytrain,'SVM','skyblue','train calibration_curve')

calibration_curve_1(1,modelXGB.predict_proba(Xtrain[f_top])[:,1],Ytrain,'KNN','deepskyblue','train calibration_curve')

plt.savefig('TrainCalibrate.tiff',dpi=1200)

##测试集校准曲线

fig = plt.gcf()

fig.set_size_inches(8,8)

# plt.subplot(1,3,2)

calibration_curve_1(1,modelLR.predict_proba(Xtest[f_top])[:,1],Ytest,'LR','red','test calibration_curve')

calibration_curve_1(1,modelBNB.predict_proba(Xtest[f_top])[:,1],Ytest,'BNB','blue','test calibration_curve')

calibration_curve_1(1,modelRF.predict_proba(Xtest[f_top])[:,1],Ytest,'RF','m','test calibration_curve')

calibration_curve_1(1,modelXGB.predict_proba(Xtest[f_top])[:,1],Ytest,'XGB','green','test calibration_curve')

calibration_curve_1(1,modelMLP.predict_proba(Xtest[f_top])[:,1],Ytest,'MLP','tomato','test calibration_curve')

calibration_curve_1(1,modelKNN.predict_proba(Xtest[f_top])[:,1],Ytest,'KNN','darkblue','test calibration_curve')

calibration_curve_1(1,modelSVM.predict_proba(Xtest[f_top])[:,1],Ytest,'SVM','skyblue','test calibration_curve')

calibration_curve_1(1,modelDT.predict_proba(Xtest[f_top])[:,1],Ytest,'DT','deepskyblue','test calibration_curve')

plt.savefig('TestCalibrate.tiff',dpi=1200)

##外部验证集校准曲线

fig = plt.gcf()

fig.set_size_inches(8,8)

# plt.subplot(1,3,3)

# calibration_curve_1(1,modelLR.predict_proba(Xtest[f_top])[:,1],Ytest,'LR','red','valid calibration_curve')

# calibration_curve_1(1,modelBNB.predict_proba(Xtest[f_top])[:,1],Ytest,'BNB','blue','valid calibration_curve')

# calibration_curve_1(1,modelRF.predict_proba(Xtest[f_top])[:,1],Ytest,'RF','m','valid calibration_curve')

# calibration_curve_1(1,modelMLP.predict_proba(Xtest[f_top])[:,1],Ytest,'MLP','tomato','valid calibration_curve')

# calibration_curve_1(1,modelKNN.predict_proba(Xtest[f_top])[:,1],Ytest,'KNN','darkblue','valid calibration_curve')

# calibration_curve_1(1,modelSVM.predict_proba(Xtest[f_top])[:,1],Ytest,'SVM','deepskyblue','valid calibration_curve')

# calibration_curve_1(1,modelDT.predict_proba(Xtest[f_top])[:,1],Ytest,'DT','deepskyblue','valid calibration_curve')

calibration_curve_1(1,modelXGB.predict_proba(Xvalid[f_top])[:,1],Yvalid,'XGB','tomato','valid calibration_curve')

plt.savefig('ValidCalibrate.tiff',dpi=1200)

#热力图

col=['XGB','RF','DT','MLP','KNN','SVM','KNN','LR','Actual']

color=['#FFFACD','#E8222D']

a=0.1

# a=Optimal_threshold(-train['OSTA_score'],train['osteoporosis'])[1]

# pre_label2=[int(-i>a) for i in train['OSTA_score'].tolist()]

tmp1=pd.DataFrame([modelXGB.predict(Xtrain[f_top]),

modelRF.predict(Xtrain[f_top]),

modelDT.predict(Xtrain[f_top]),

modelMLP.predict(Xtrain[f_top]),

modelKNN.predict(Xtrain[f_top]),

modelSVM.predict(Xtrain[f_top]),

modelKNN.predict(Xtrain[f_top]),

modelLR.predict(Xtrain[f_top]),

# DecisionTree.predict(train[f_top]),

# rf.predict(train[f_top]),

# lr.predict(train[f_top]),

# mlp.predict(train[f_top]),

# BNB.predict(train[f_top]),

# pre_label2,

Ytrain],index=col).T

fig = plt.gcf() # gcf: get current figure调整图形大小

fig.set_size_inches(20,12)

plt.subplot(1,2,1)

sns.heatmap(data=tmp1,

vmax=1,

vmin=0,cmap = color,yticklabels=False,cbar=False

# cmap='YlOrRd_r',

# annot=True,

# fmt=".3f",

)

# sns.cubehelix_palette(as_cmap=True, reverse=True)

plt.title('Training set')

# plt.ylabel('N=%s'%Xtrain.shape[0],fontsize=20, color='k') #y轴label的文本和字体大小

plt.ylabel("N=19026",fontsize=20, color='k') #y轴label的文本和字体大小

plt.subplot(1,2,2)

col=['XGB','RF','DT','MLP','KNN','SVM','KNN','LR','Actual']

color=['#FFFACD','#E8222D']

a=0.1

# a=Optimal_threshold(-train['OSTA_score'],train['osteoporosis'])[1]

# pre_label2=[int(-i>a) for i in train['OSTA_score'].tolist()]

tmp2=pd.DataFrame([modelXGB.predict(Xtest[f_top]),

modelRF.predict(Xtest[f_top]),

modelDT.predict(Xtest[f_top]),

modelMLP.predict(Xtest[f_top]),

modelKNN.predict(Xtest[f_top]),

modelSVM.predict(Xtest[f_top]),

modelKNN.predict(Xtest[f_top]),

modelLR.predict(Xtest[f_top]),

# DecisionTree.predict(train[f_top]),

# rf.predict(train[f_top]),

# lr.predict(train[f_top]),

# mlp.predict(train[f_top]),

# BNB.predict(train[f_top]),

# pre_label2,

Ytest],index=col).T

fig = plt.gcf() # gcf: get current figure调整图形大小

fig.set_size_inches(12,12)

sns.heatmap(data=tmp2,

vmax=1,

vmin=0,cmap = color,yticklabels=False,cbar=False

# cmap='YlOrRd_r',

# annot=True,

# fmt=".3f",

)

# sns.cubehelix_palette(as_cmap=True, reverse=True)

plt.title('Testing set')

# plt.ylabel('N=%s'%Xtrain.shape[0],fontsize=20, color='k') #y轴label的文本和字体大小

plt.ylabel("N=8154",fontsize=20, color='k') #y轴label的文本和字体大小

##验证集热力图

plt.subplot(1,3,3)

col=['XGB','RF','DT','MLP','KNN','SVM','KNN','LR','Actual']

color=['#FFFACD','#E8222D']

a=0.1

tmp3=pd.DataFrame([modelXGB.predict(Xvalid[f_top]),

modelRF.predict(Xvalid[f_top]),

modelDT.predict(Xvalid[f_top]),

modelMLP.predict(Xvalid[f_top]),

modelKNN.predict(Xvalid[f_top]),

modelSVM.predict(Xvalid[f_top]),

modelKNN.predict(Xvalid[f_top]),

modelLR.predict(Xvalid[f_top]),

# DecisionTree.predict(train[f_top]),

# rf.predict(train[f_top]),

# lr.predict(train[f_top]),

# mlp.predict(train[f_top]),

# BNB.predict(train[f_top]),

# pre_label2,

Yvalid],index=col).T

fig = plt.gcf() # gcf: get current figure调整图形大小

fig.set_size_inches(20,12)

sns.heatmap(data=tmp3,

vmax=1,

vmin=0,cmap = color,yticklabels=False,cbar=False

# cmap='YlOrRd_r',

# annot=True,

# fmt=".3f",

)

# sns.cubehelix_palette(as_cmap=True, reverse=True)

plt.title('Valid set')

# plt.ylabel('N=%s'%Xtrain.shape[0],fontsize=20, color='k') #y轴label的文本和字体大小

plt.ylabel("N=1118",fontsize=20, color='k') #y轴label的文本和字体大小

plt.savefig('ActualHeatmap.tiff',dpi=1200)

###SHAP图

fig = plt.gcf()

fig.set_size_inches(8,8)

explainer = shap.TreeExplainer(modelXGB)

shap_values = explainer.shap_values(Xtrain)

shap.summary_plot(shap_values,Xtrain)

plt.savefig('shap_summary_plot.tiff', dpi=300, format='tiff')

#特征交互式解释

shap_interaction_values = explainer.shap_interaction_values(Xtrain)

shap.summary_plot(shap_interaction_values, Xtrain)

shap.force_plot(explainer.expected_value, shap_values[10, :], X.iloc[10, :], matplotlib=True)

shap.force_plot(explainer.expected_value, shap_values[100, :], X.iloc[100, :], matplotlib=True)
